# Multiple σ^EcfG^ and NepR Proteins Are Involved in the General Stress Response in *Methylobacterium extorquens*

**DOI:** 10.1371/journal.pone.0152519

**Published:** 2016-03-30

**Authors:** Anne Francez-Charlot, Julia Frunzke, Judith Zingg, Andreas Kaczmarczyk, Julia A. Vorholt

**Affiliations:** Institute of Microbiology, ETH Zurich, Zurich, Switzerland; University Medical Center Utrecht, NETHERLANDS

## Abstract

In Alphaproteobacteria, the general stress response (GSR) is controlled by a conserved partner switch composed of the sigma factor σ^EcfG^, its anti-sigma factor NepR and the anti-sigma factor antagonist PhyR. Many species possess paralogues of one or several components of the system, but their roles remain largely elusive. Among Alphaproteobacteria that have been genome-sequenced so far, the genus *Methylobacterium* possesses the largest number of σ^EcfG^ proteins. Here, we analyzed the six σ^EcfG^ paralogues of *Methylobacterium extorquens* AM1. We show that these sigma factors are not truly redundant, but instead exhibit major and minor contributions to stress resistance and GSR target gene expression. We identify distinct levels of regulation for the different sigma factors, as well as two NepR paralogues that interact with PhyR. Our results suggest that in *M*. *extorquens* AM1, *ecfG* and *nepR* paralogues have diverged in order to assume new roles that might allow integration of positive and negative feedback loops in the regulatory system. Comparison of the core elements of the GSR regulatory network in *Methylobacterium* species provides evidence for high plasticity and rapid evolution of the GSR core network in this genus.

## Introduction

The general stress response (GSR) is a widely conserved response in bacteria for environmental adaptation. It is defined as a preventive response induced by diverse stressful conditions and is generally governed by complex regulatory systems [1–3]. In Alphaproteobacteria, the GSR is controlled by a sigma factor of the ECF (extracytoplasmic function) family generally called σ^EcfG^ [4]. The activity of σ^EcfG^ is regulated by the anti-sigma factor NepR and the anti-sigma factor antagonist PhyR. The three proteins function in a partner switch: in absence of stress, σ^EcfG^ is sequestered by NepR while PhyR is inactive. In response to stress, PhyR becomes phosphorylated and binds NepR, releasing σ^EcfG^ that can then associate with the RNA polymerase core enzyme to transcribe stress-related genes (for review, [1, 5]). HRXXN histidine kinases/phosphatases control PhyR phosphorylation, the number of which varies in different Alphaproteobacteria, as do the signaling domains they harbor, presumably as a consequence of the different conditions activating the GSR in various species [6–11]. Variations are also observed in the genes regulated in different organisms, which probably reflects the requirements and physiology imposed by specific niches colonized by distinct species [1]. PhyR, NepR and σ^EcfG^ are usually encoded at the same genetic locus, but paralogues of the respective genes are often also found elsewhere in the genome of a given organism [1, 12], and little is known about the roles of these paralogues. The only organism studied so far for the roles of multiple *phyR* and *nepR* genes is *Sinorhizobium meliloti*, which possesses two PhyR (RsiB1 and RsiB2) and two NepR (RsiA1 and RsiA2) proteins. Both RsiA anti-sigma factors interact *in vitro* with RpoE2 (σ^EcfG^ orthologue) and with both RsiB proteins [13]. *In vivo*, RsiB1 and RsiB2 have redundant activities and both RsiA1 and RsiA2 act as negative regulators of the cascade. However, only the deletion of *rsiA1* is lethal (being toxic due to the overactivation of the cascade), indicating that the function of these paralogues are not fully redundant [13]. Differences are also observed in their regulation, as, contrary to *rsiA1*, *rsiA2* is not regulated by RpoE2 [13]. It is currently not known which features are provided to GSR regulation by these additional regulators in *S*. *meliloti*. The presence of additional copies of *ecfG* is more common [1, 12] but only a few studies on their functions have been reported. *Caulobacter crescentus* and *Sphingomonas melonis* Fr1 possess a paralogue of *ecfG1* (*sigT* in *C*. *crescentus*), *sigU* and *ecfG2*, respectively. In both cases, *sigT*/*ecfG1* deletion has dramatic effects on stress resistance, whereas no obvious defect is associated with *sigU*/*ecfG2* deletion [9, 10, 14]. In addition, *sigU*/*ecfG2* expression is regulated at the transcriptional level by SigT/σ^EcfG1^, and SigU/σ^EcfG2^ does not interact with NepR [9, 10, 14]. In *C*. *crescentus*, it was shown that SigU does not possess a separate regulon but controls the expression of several SigT-regulated genes, and was thus proposed to provide mild amplification of the GSR [9]. In contrast, in *Rhizobium etli*, which also possesses two *ecfG* paralogues, *ecfG1* and *ecfG2*, the situation appears to be different; first, the expression of *ecfG2* is only partially dependent on *ecfG1* [15]; and second, *ecfG1* and *ecfG2* mutants exhibit different phenotypes and regulate both common and distinct targets [15]. Possible interactions between the sigma factors and NepR, or NepR and PhyR, were not tested and their functional role in the GSR is currently unclear. In sum, the role of *ecfG2* (*sigU* in *C*. *crescentus*) appears to be different among alphaproteobacterial species, an observation that might also be linked to the fact that these paralogues likely originated from independent, lineage-specific duplication events of *ecfG1* [15].

Lineage-specific expansion of regulatory components of the GSR can be more pronounced: *Methylobacterium* strains genome-sequenced to date possess up to eleven *ecfG* genes. Their contribution to the GSR has not been systematically assessed. It is known, however, that several of them must be involved in the GSR, since deletion of *ecfG1*, one of the six *ecfG* paralogues of *M*. *extorquens* AM1, does not lead to stress sensitivity, in contrast to the increased stress sensitivity of a *phyR* mutant [4]. In order to assign roles for the σ^EcfG^ homologues of *M*. *extorquens* AM1 and to understand the apparent complexity of the cascade, in this study we investigated their contributions to stress resistance, their interactions and their regulation.

## Materials and Methods

### Bacterial strains and growth conditions

Strains used in this study are listed in S4 Table. *M*. *extorquens* AM1 strains were grown at 28°C on minimal medium (MM) [16] with a modified trace elements solution [17] and supplemented with 120 mM methanol. *E*. *coli* DH5α, TOP10 (Invitrogen) or a *dam*^-^ *dcm*^-^ strain (NEB) were used for cloning purposes, and *E*. *coli* BTH101 (Euromedex) for bacterial two-hybrid assays. *E*. *coli* strains were grown on LB at 37°C for cloning or at 28°C for bacterial two-hybrid assays. When appropriate, the media were supplemented with kanamycin (50 μg/ml), tetracycline (10 μg/ml), and carbenicillin (50 μg/ml).

If not otherwise stated, for all experiments with *M*. *extorquens* AM1 (phenotypes, co-immunoprecipitation, measurement of luciferase activity and Western blots), precultures were inoculated from plate and grown on minimal medium for 8-24h at 28°C. The main cultures were inoculated from precultures to an optical density at 600 nm (OD_600_) of 0.04 to 0.08 and grown on MM at 28°C until an OD_600_ of 1 was reached.

### Plasmid and strain construction

Plasmids used in this study are listed in S4 Table and primers in S5 Table. All DNA manipulations were performed according to standard protocols [18].

For generation of in-frame deletion mutants in *M*. *extorquens* AM1, a *sacB*-based allelic exchange method using pCM433 [19] or pK18mobsacB [20] was employed. For complementation purposes, pCM62 or pCM80 were used [21]. For production of C-terminal HA-tagged σ^EcfG^ or C-terminal triple FLAG-tagged NepR, pCM62HA and pCM80_3xF were used, respectively. pCM62HA is a derivative of pCM62 and was constructed as follows: primers HA_s and HA_as were annealed and cloned into the EcoRI/Acc65I sites of pCM62. To construct pCM80_3xF, a fragment corresponding to the triple FLAG tag-encoding sequence was amplified by PCR using primers Flag F and 3xR, the product was digested with Acc65I/EcoRI and cloned into the same sites of pCM80. For transcriptional fusions, putative promoter regions (ca. 200 bp upstream to 30 bp downstream of the start codon) of the genes of interest were cloned into the plasmid pLM05 [22], which contains the promoter-less *luxCDABE* operon of *Photorhabdus luminescens*. All plasmids were introduced by electroporation in *M*. *extorquens* AM1. For bacterial two-hybrid assays, *nepR* and *ecfG* paralogues were cloned in pUT18 and pKNT25, respectively, of the bacterial two-hybrid system [23].

### Sequence and phylogenetic analyses

Phylogenetic analyses were conducted in MEGA6 [24]. Sequences were aligned with ClustalW with MEGA6 default parameters [25]. The evolutionary history was inferred using the Neighbor-Joining method [26]. Bootstrap tests were performed with 500 replicates [27]. The evolutionary distances were computed using the Poisson correction method [28] and are in the units of the numbers of amino acid substitutions per site. All positions with less than 95% site coverage were eliminated. That is, fewer than 5% alignment gaps, missing data, and ambiguous residues were allowed at any position.

To search for σ^EcfG^ paralogues in *Methylobacterium* spp., the protein sequence of σ^EcfG1^ of *M*. *extorquens* AM1 was used as query in a BLAST search on the JGI website (https://img.jgi.doe.gov/cgi-bin/edu/main.cgi). Proteins were considered as σ^EcfG^ paralogues if the score and E-value of the BLAST hits were at least 165 bits and 1e-50, respectively, except for Mext_0132, which gave a score of 134 and an E-value of 6e-39, since the protein only consists of sigma factor region σ_2_. Note that sigma factors of other subfamilies gave scores below 106 and E-values below 1e-30, defining a clear cutoff between the ECF15 subfamily and other ECF sigma factor groups.

### Phenotypic assays

Sensitivity to methylglyoxal was tested in disk diffusion assays as previously described [29]. To test sensitivity to salts or ethanol, ten-fold serial dilutions of cultures were spotted onto MM plates supplemented with methanol and containing 100 mM NaCl or 1.5% ethanol. Three biological replicates were performed for each experiment, and the mean +/- SD or one representative experiment is shown.

### RNA preparation

Cultures (20 ml) started from fresh plates were grown for 8–10 h, diluted to an OD_600_ of 0.04 (50 ml) and incubated ON at 28°C. Cultures were split in two (20 ml each) and supplemented with ethanol (1% final), and further incubated for 20 min at 28°C. A solution of 5% phenol in ethanol was added (10% final) and cultures were spun down and the pellets were flash-frozen in liquid nitrogen and kept at -80°C until further processed. Three biological replicates were made for each condition. RNA was extracted with the RNeasy kit (Qiagen) following the manufacturer’s instructions. After purification, RNA samples were treated twice with DNase (Ambion). Absence of DNA was verified by PCR and RNA integrity was checked after denaturation on agarose gels.

### Microarrays analyses

The 60-mer oligonucleotide custom-designed Agilent arrays for *M*. *extorquens* (Santa Clara, CA) were described previously [30]. cDNA production and labeling, hybridization and basic analyses were performed by MOgene (St. Louis, MO). For the basic analyses, data were extracted using Agilent Feature Extraction, including LOWESS dye normalization. Data were then processed in GeneSpring 12.5 with the following sequence: thresholding, summarization, log transformation and percentile normalization. An unpaired t-test was performed on normalized values in Matlab and genes with a p-value of less than 0.005 and an absolute fold-change of at least 2 were considered significantly differentially expressed. Hierarchical cluster analysis and heat map generation were done in MeV [31, 32]. Hierarchical cluster analysis was performed using MeV standard settings.

Microarray data are available in the ArrayExpress database (www.ebi.ac.uk/arrayexpress) under accession number E-MTAB-3461.

### Motif discovery using MEME

Upstream regions of differentially expressed genes were subjected to a motif search using MEME [33] with the following relevant parameters: distribution of motif occurrences, 0 or 1 per sequence; number of different motifs, 5; minimum motif width, 25; maximum motif width, 40; searching, “given strand only”. Sequences with putative σ^EcfG^-type promoters were downloaded and alignments were manually adjusted to account for the variable spacer region (16 or 17 nucleotides) between the -10 and -35 boxes. Refined alignments were used to generate a consensus sequence using the Weblogo tool [34].

### Bacterial two-hybrid assay

Plasmids were transformed sequentially or co-transformed in *E*. *coli* BTH101. The transformants were plated on LB containing appropriate antibiotics and supplemented with 1 mM IPTG and 40 μg/ml X-Gal. Blue coloration was evaluated after two days of incubation at 28°C. To measure β-galactosidase activity, liquid cultures were inoculated from single colonies and grown overnight in LB containing appropriate antibiotics and supplemented with 1 mM IPTG. β-galactosidase assays were performed as described previously [35].

### Measurement of luciferase activity

Aliquots of 90 μl were taken from bacterial cultures (20 ml) at an OD_600_ of 1 and mixed with 10 μl of sterile deionized water (control), 10% ethanol or 200 mM NaCl in 96-well B&W isoplates (PerkinElmer). Luminescence was measured 99 times during 120 min using the VICTOR^3^ multiple plate reader (PerkinElmer Life and Analytical Sciences), and time points shown in the figures correspond to maximum induction after approximately 50–60 min. For measurement of stationary phase promoter activity, the same 20-ml cultures were grown for another 20 h before luciferase activity was measured. Luciferase activities are expressed as counts per second (CPS) per OD_600_ in arbitrary units (AU). Three biological replicates were performed for each experiment and means +/- SD are shown.

### Determination of protein levels by Western blotting

Cultures at an OD_600_ of 1 were treated with ethanol or NaCl at a final concentration of 1% and 20 mM, respectively, and incubated with shaking for 20 min at 28°C. Bacteria were harvested by centrifugation and resuspended in SDS-PAGE loading buffer. The samples were subjected to 12.5% SDS-PAGE, transferred to a nitrocellulose membrane using semidry blotting, and immunoblotted with primary rabbit anti-HA antibodies (Sigma-Aldrich) and secondary goat anti-rabbit IgG-HRP-conjugated antibodies (Biorad).

### Co-immunoprecipitation

Cells from a 100-ml culture at an OD_600_ of 1 were harvested by centrifugation, washed once in 10 ml TBS buffer (50 mM Tris-HCl pH 7.4; 150 mM NaCl) and resuspended in 0.5 ml of TBS supplemented with protease inhibitor (Complete EDTA-free, Roche) and lysozyme (1 mg/ml). Bacteria were lysed by beating with 300 μl of zirconia beads (BioSpec) in a 3M ESPE CapMix instrument. After centrifugation and DNase addition, the supernatants were incubated for 1 h at room temperature with 40 μl of anti-FLAG M2 affinity gel (Sigma-Aldrich). The resin was washed 4 times with 0.5 ml of TBS supplemented with protease inhibitor before elution with 40 μl of 2-fold non-reducing SDS-PAGE loading buffer. The fractions were separated on a 15% SDS-PAGE gel, transferred to a nitrocellulose membrane and immunoblotted with rabbit anti-PhyR serum (Neoclone) or mouse anti-FLAG M2 primary antibodies (Sigma-Aldrich) and alkaline phosphatase-conjugated goat anti-rabbit or goat anti-mouse secondary antibodies (Biorad).

## Results

### Features of *M*. *extorquens* AM1 σ^EcfG^ proteins and their encoding genes

*M*. *extorquens* AM1 possesses 14 sigma factors, six of which belong to the ECF15 (or σ^EcfG^) subfamily of ECF sigma factors (S1 Table, [36]). In an analysis of phylogenetic relationships of σ^EcfG^ sigma factors, *M*. *extorquens* AM1 σ^EcfG^ proteins appeared more closely related to each other than to σ^EcfG^ proteins of other members of the Rhizobiales, suggesting that they are paralogous (S1 Fig).

In general, *ecfG*, *nepR* and *phyR* are found at the same locus in Alphaproteobacteria. Most often, *ecfG* and *nepR* form an operon that is divergently transcribed from *phyR*, and both transcriptional units are under the control of σ^EcfG^-dependent promoters. *M*. *extorquens* AM1 represents an exception, since none of the six *ecfG* genes is located at the *phyR* locus. Furthermore, no putative σ^EcfG^-type promoter with the consensus GAAC-N_16,17_-C/GGTT is found in the upstream region of these genes, suggesting none of them is autoregulated.

Another peculiarity of σ^EcfG^ proteins of *M*. *extorquens* AM1 is an N-terminal extension of about 55 amino acids not present in other Alphaproteobacteria except in the Methylobacteriaceae. As noted before [4], this extension resembles NepR (S2A and S2B Fig), suggesting it might be the result of an ancient fusion event between an *ecfG* and a *nepR* gene. Whether this extension provides additional features to the sigma factors is currently not known.

We previously detected two versions of σ^EcfG1^ *in vivo*, the full-length form and a shorter version missing the first 14 residues [4]. Mutational analysis indicated that the shorter isoform results from translation initiation from a second start codon (S3A and S3B Fig). Both σ^EcfG1^ isoforms could complement a sextuple *ecfG* mutant (see below and S3C Fig) and could interact with NepR *in vivo* as judged by pull down experiments [4], and it is currently not clear whether the isoforms have different roles. The other σ^EcfG^ proteins did not appear to exist as multiple isoforms under the conditions tested (see below).

### Phenotypic analysis of *ecfG* mutants identifies two major σ^EcfG^ proteins

**Fig 1:**
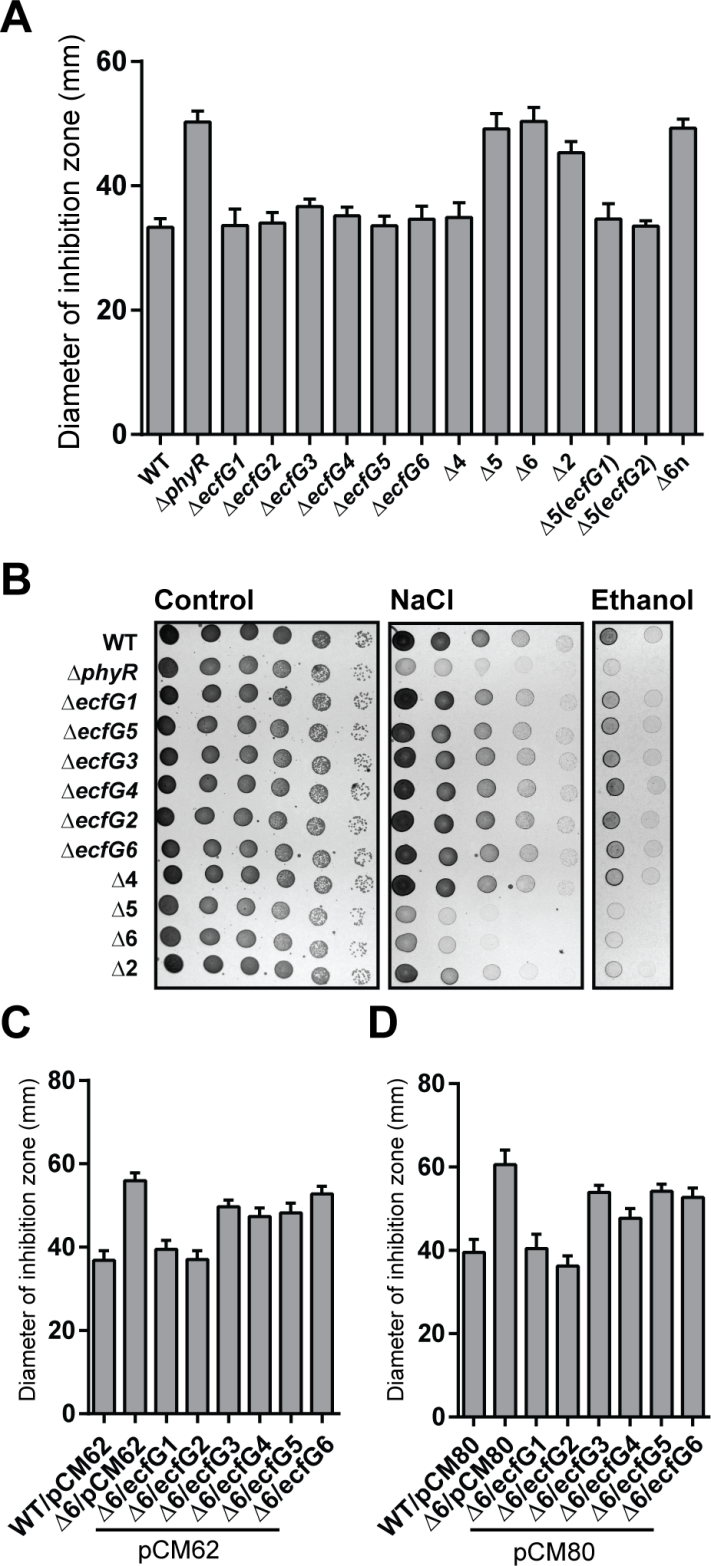
Phenotypic analysis of *ecfG* mutants. A. Sensitivity to methylglyoxal of the wild type (WT), *phyR* mutant (Δ*phyR*), *ecfG* single mutants (Δ*ecfG1*, Δ*ecfG2*, Δ*ecfG3*, Δ*ecfG4*, Δ*ecfG5*, and Δ*ecfG6*) and *ecfG* multiple mutants (Δ4, Δ5, Δ2, Δ5(*ecfG1*), Δ5(*ecfG2*), Δ6n). B. Growth of the same strains on MM supplemented with NaCl (middle panel) or ethanol (right panel) compared to growth on MM (left panel). Ten-fold serial dilutions are shown from left to right, starting from undiluted samples. C and D. Sensitivity to methylglyoxal of the sextuple mutant expressing each *ecfG* from its own promoter on pCM62 (C) or from the strong *mxaF* promoter on pCM80 (D).

We previously demonstrated the involvement of *ecfG1* in the GSR of *M*. *extorquens* AM1: σ^EcfG1^ was shown to regulate a subset of the PhyR regulon and to interact with NepR [4]. Despite these observations, an *ecfG1* mutant does not exhibit stress sensitivity, in contrast to a *phyR* mutant, suggesting that other σ^EcfG^ proteins are involved in the GSR. To test this hypothesis, we constructed single and multiple *ecfG* mutants and analyzed their sensitivity to different stresses. As shown in Fig 1A and 1B, no increased sensitivity to methylglyoxal, salt or ethanol was observed for any single mutant or for the quadruple mutant (Δ4, corresponding to Δ*ecfG1* Δ*ecfG3* Δ*ecfG4* Δ*ecfG5*) compared to the wild-type strain. In contrast, the quintuple mutant (Δ5, corresponding to Δ*ecfG1* Δ*ecfG3* Δ*ecfG4* Δ*ecfG5* Δ*ecfG2*) was more sensitive than the wild type to methylglyoxal, salt and ethanol, to the same extent as a sextuple mutant (Fig 1A and 1B). Since the difference between the Δ4 and Δ5 strains is the deletion of *ecfG2*, we tested whether an *ecfG1 ecfG2* double mutant (Δ2) was stress sensitive. Indeed, this mutant showed increased sensitivity to all stresses tested compared to the wild type, albeit the strain was not as stress sensitive as the quintuple and sextuple mutants (Fig 1A and 1B). To rule out that the combination of deletions of one or several of the four remaining *ecfG* genes together with the deletion of either *ecfG1* or *ecfG2* is sufficient to confer stress sensitivity, a Δ*ecfG3* Δ*ecfG4* Δ*ecfG5* Δ*ecfG6* deletion mutant (Δ4n) was first constructed, followed by deletion of *ecfG1* or *ecfG2*. None of these quintuple mutants [Δ5(*ecfG1*) and Δ5(*ecfG2*)] showed increased stress sensitivity. Only deletion of both *ecfG1* and *ecfG2* in the Δ4n strain (Δ6n) resulted in a stress sensitive strain (Fig 1A), indicating that deletion of either *ecfG1* or *ecfG2* in combination with the remaining *ecfG* genes is not sufficient to confer stress sensitivity.

We next tested whether each σ^EcfG^ could individually complement the sextuple mutant. To this end, *ecfG* open reading frames including 200bp upstream of the start codon were cloned in plasmid pCM62 and the resulting plasmids were introduced in the Δ6 strain (Fig 1C). When sensitivity to methylglyoxal was tested, only expression of *ecfG1* or *ecfG2* resulted in full complementation. For the other *ecfG* paralogues, no complementation was observed. Essentially the same results were obtained when each *ecfG* gene was expressed individually from the strong *mxaF* promoter, except for *ecfG4* which could partially complement (Fig 1D). This suggests, except for *ecfG4*, that the failure of *ecfG* genes to complement when cloned in pCM62 was not due to the absence of expression.

Altogether, these data suggest that *ecfG1* and *ecfG2* have redundant major roles in the GSR of *M*. *extorquens* AM1. The remaining four σ^EcfG^ proteins also contribute to stress resistance, but apparently to a smaller extent.

### Microarray analysis of *ecfG* mutants confirms the major roles of σ^EcfG1^ and σ^EcfG2^

**Fig 2:**
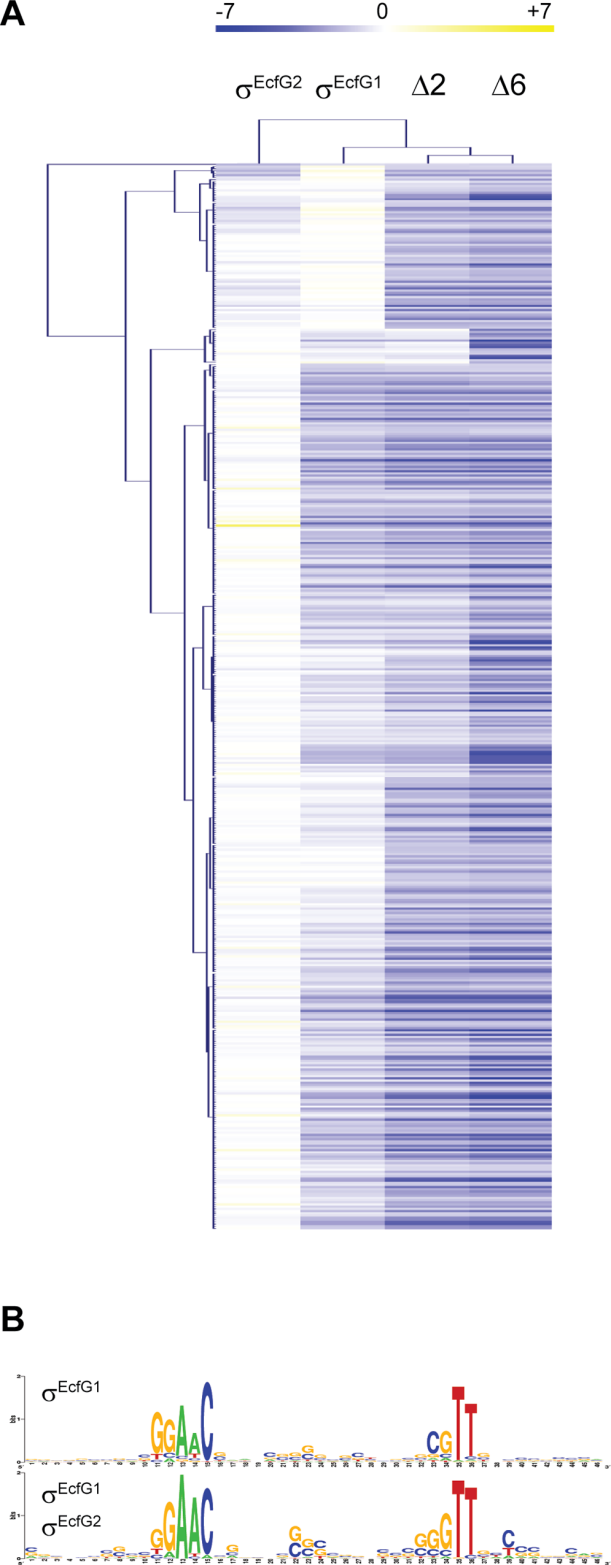
Genes controlled by σ^EcfG^ proteins. (A) Heat map visualization of hierarchical cluster analysis of the log_2_ fold-change of the 490 genes downregulated in the Δ6 strain in all *ecfG* mutant backgrounds. The genes are represented in rows and the mutant backgrounds in column. (B) Weblogo output of the motifs found by MEME.

The results described above suggest that σ^EcfG^ proteins of *M*. *extorquens* AM1 work in parallel to activate the expression of their target genes to ultimately confer stress resistance. More specifically, two mechanisms could be envisaged to explain the apparent functional redundancy of σ^EcfG^ proteins: (i) they regulate the same set of genes and the resulting phenotype is due to dosage effects; or (ii) different sets of genes that have redundant functions in stress resistance are controlled by distinct σ^EcfG^ proteins. In order to test these hypotheses, we used microarrays to identify genes differentially expressed between wild-type and Δ*ecfG1*, Δ*ecfG2*, Δ2 or Δ6 strains after addition of ethanol. 490 genes were downregulated at least 2-fold (p-value < 0.005) in the Δ6 strain compared to the wild type (S2 Table). Note that only downregulated genes were considered, in order to limit the number of indirect target genes. Since the genes downregulated in the Δ6 strain should comprise all genes differentially expressed in all strains and conditions tested, we looked at the average fold-changes of these genes in the other genetic backgrounds to determine on which σ^EcfG^ protein their expression depended. Most genes differentially regulated in the Δ6 strain were also differentially regulated in the *ecfG1 ecfG2* double mutant (87%), with about half already differentially expressed in the *ecfG1* mutant (S2 Table). In contrast, only twelve genes were differentially regulated in the *ecfG2* mutant. These results suggest that most GSR target genes are dependent on either σ^EcfG1^ or σ^EcfG2^. While the data suggest that σ^EcfG1^ possesses its own regulon, the fact that many target genes showed higher average fold-changes in the *ecfG1 ecfG2* double mutant compared to the single mutants suggests contribution of both sigma factors (Fig 2A). Our data also indicate that at least 66 genes might be controlled by one or several σ^EcfG^ proteins other than σ^EcfG1^ or σ^EcfG2^ or in addition to σ^EcfG1^ or σ^EcfG2^. In fact, comparing the fold-changes of “Δ6 vs WT” and “Δ2 vs WT” experiments suggests that such combinatorial regulation might occur for more than these 66 GSR targets (Fig 2A).

Next, we analyzed the promoter regions of GSR target genes. Putative promoter sequences of genes downregulated (i) in the *ecfG1* mutant, (ii) in the Δ2 strain but not in the *ecfG1* mutant, and (iii) in the Δ6 but not in the Δ2 strain, were analyzed separately for the presence of enriched motifs. 136 out of 223 σ^EcfG1^ targets (61%) and 55 out of 200 σ^EcfG1^/σ^EcfG2^ targets (27.5%) harbored a putative σ^EcfG^-type promoter with the consensus GAAC-N_16,17_-C/GGTT (Fig 2B), whereas no σ^EcfG^-type promoter was found as enriched motif in the third group. These data suggest that σ^EcfG^ sigma factors of *M*. *extorquens* AM1 recognize similar promoters, in agreement with the conservation of their σ_4.2_ and σ_2.4_ regions, responsible for binding the -35 and -10 boxes of the promoter, respectively (S2A Fig). The absence of σ^EcfG^-type promoters in 299 genes of the regulon might be partially explained by the existence of operons, which were not taken into account in the analysis; the others genes might represent indirect targets controlled by transcriptional regulators themselves targets of σ^EcfG^ sigma factors (see S2 Table).

In conclusion, our data suggest that σ^EcfG2^ does not possess its own regulon, but instead controls the same set of target genes as σ^EcfG1^. These results are in agreement with the hypothesis that the stress sensitivity of the *ecfG1 ecfG2* double mutant reflects a dosage effect on GSR target genes expression rather than control of distinct functionally redundant regulons.

### GSR induction upon acute stress depends on σ^EcfG1^

**Fig 3:**
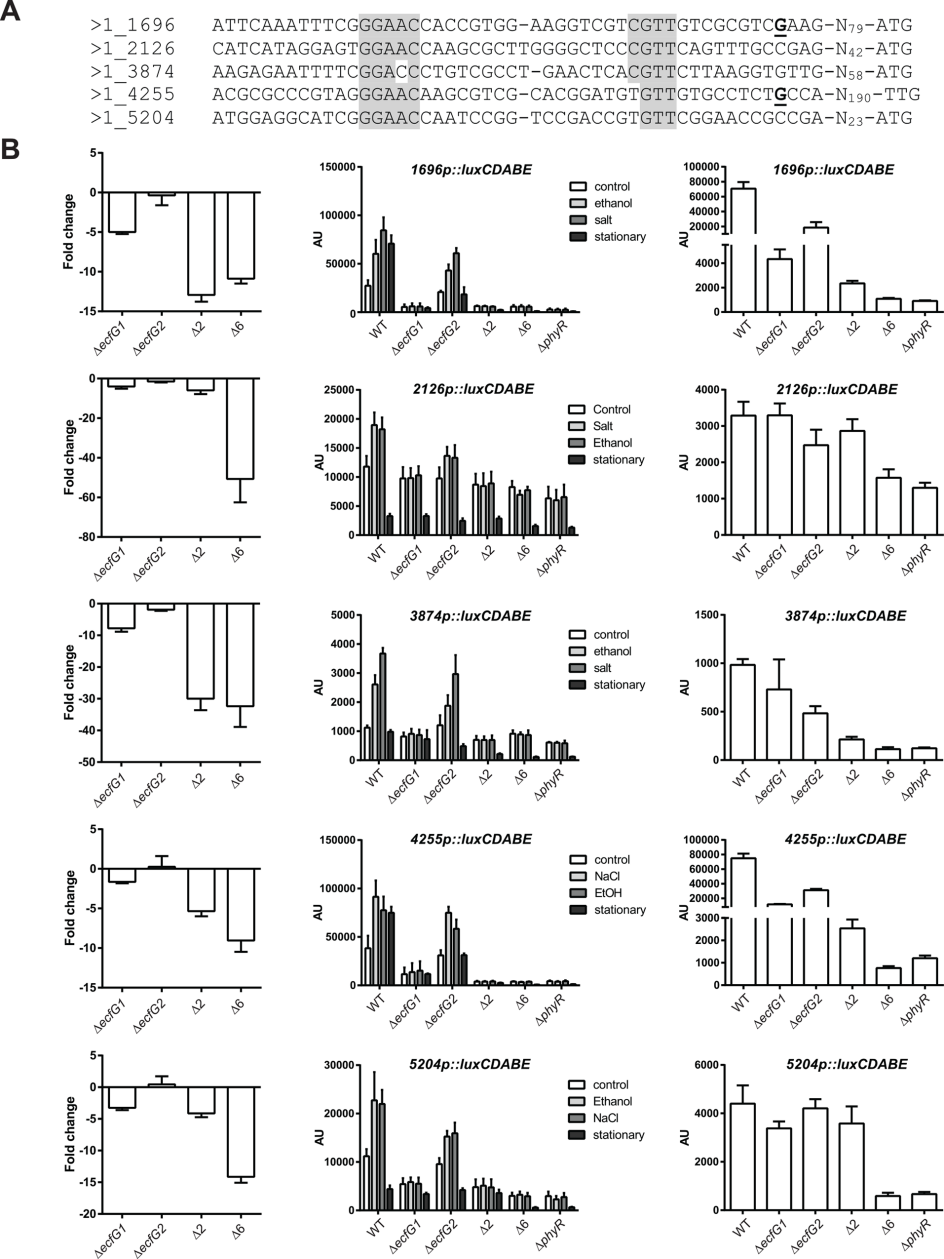
Activity of selected promoters in response to salt, ethanol or in stationary phase. A. Sequences of selected promoters. The transcriptional start sites mapped previously are in bold and underlined [22, 29]. The putative -35 and -10 boxes are highlighted in grey. B. (left panel) Fold-change values from microarray experiments. (middle panel) Luciferase activity of *luxCDABE* transcriptional fusions to selected promoters in the wild type (WT), *ecfG1* mutant (Δ*ecfG1*), *ecfG2* mutant (Δ*ecfG2*), double *ecfG1 ecfG2* mutant (Δ2), sextuple mutant (Δ6) or *phyR* mutant (Δ*phyR*). Cultures were treated with 1% ethanol (ethanol), 20 mM salt (salt) or H_2_O (control) in exponential phase 60 minutes prior to measurement, or were grown to stationary phase. The right panel shows the same stationary phase values with different axis ranges in order to better see the differences between the strains. AU, arbitrary units.

To confirm the microarray results, we analyzed five target promoters (Fig 3A) using *luxCDABE* transcriptional fusions in the different backgrounds in response to ethanol and salt treatment, in order to test whether the same induction patterns were observed for another stress. Based on microarray results, the promoters chosen seemed to depend only on σ^EcfG1^, or σ^EcfG1^ and σ^EcfG2^, with or without contribution of the remaining σ^EcfG^ proteins (Fig 3B, left panels). All five promoters tested were inducible in exponential phase by salt or ethanol in the wild-type strain (Fig 3B, middle panel), and induction was abolished in the Δ*ecfG1*, Δ2, Δ6 or Δ*phyR* backgrounds, but not in the Δ*ecfG2* background, although induction in the latter background appeared reduced compared to the wild type (Fig 3B, middle panel). In exponential phase without stress, the basal luciferase activity of the fusions was not changed in the different backgrounds compared to the wild type for *2126p* and *3874p*, whereas *1696p*, *4255p*, *5204p* showed lower activity in Δ*ecfG1*, Δ2, Δ6 or Δ*phyR* backgrounds (Fig 3B, middle panel). Thus, in exponential phase, contributions of σ^EcfG1^ and σ^EcfG2^ but not of the other σ^EcfG^ proteins to the expression of the different genes were observed. Since the contribution of the minor σ^EcfG^ sigma factors was not evident in response to acute stress for the promoters tested, we sought to analyze the response in stationary phase. In this condition, *1696p* and *4255p* were induced, whereas activity of the other promoters was the same as in exponential phase or even reduced (Fig 3B, middle panel). Importantly, for all promoters tested, the expression levels were lower in the Δ6 strain compared to the Δ2 strain (Fig 3B, right panel). Altogether, these data suggest that each target is controlled by a combination of several σ^EcfG^ sigma factors, with σ^EcfG1^ playing a major role in activation of gene expression in response to acute stress. The finding that σ^EcfG^ contribution apparently depends on the conditions raises the possibility that different σ^EcfG^ sigma factors are regulated in response to different signals. We next explored how the σ^EcfG^ proteins could be regulated.

### σ^EcfG1^ and σ^EcfG5^ interact with the anti-sigma factor NepR

**Fig 4:**
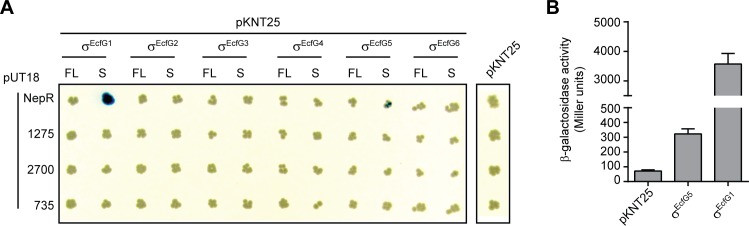
Interactions between σ^EcfG^ and NepR paralogues. A. Interactions between NepR or its paralogues and full-length (FL) or N-terminal truncated version (S) of each σ^EcfG^ were tested in the bacterial two-hybrid system. Blue coloration indicates interaction. B. Beta-galactosidase activity of the strains carrying the plasmid pUT18-nepR, and pKNT25-ecfG1, pKNT25-ecfG5 or the empty pKNT25 plasmid. The values are given in Miller units as means +/- SD of three biological replicates.

It has previously been established that σ^EcfG1^ is controlled by the anti-sigma factor NepR and the anti-sigma factor antagonist PhyR in a partner switch [4]. To test whether the σ^EcfG^ paralogues are also regulated in the same way, interactions between NepR and all σ^EcfG^ proteins were analyzed in a bacterial two-hybrid assay [23]. Two versions were examined for each σ^EcfG^, with (FL) or without (S) their NepR-like N-terminal extension, since only a σ^EcfG1^ version truncated of its N-terminal extension is able to bind NepR *in vitro* (Fig 4A; [4]). Two interactions were detected: a strong interaction between the truncated version of σ^EcfG1^ and NepR, as expected, and a weaker interaction between the truncated version of σ^EcfG5^ and NepR (Fig 4A and 4B). Comparing the residues of *M*. *extorquens* σ^EcfG^ proteins presumed to interact with NepR based on the homology model of NepR-σ^EcfG^ of *S*. *melonis* Fr1 [37] indicate that most positions are conserved between σ^EcfG1^ and the other σ^EcfG^ proteins, except for σ^EcfG2^, for which several positions are substituted (S2A Fig).

Given the absence of detectable interactions between most σ^EcfG^ proteins and NepR, we wondered whether NepR homologues might exist and thus searched the *M*. *extorquens* genome. BLAST/PSI-BLAST searches identified three putative NepR homologues (MexAM1_META1p1275, MexAM1_META1p2700 and MexAM1_META2p0735) (see S2C Fig) and possible interactions between each σ^EcfG^ and each NepR protein were analyzed in the bacterial two-hybrid system. None of the putative NepR homologues interacted with any σ^EcfG^ proteins (Fig 4A). Although we cannot exclude the possibility that the experimental system failed to detect interactions, these data suggest that, except σ^EcfG1^ and σ^EcfG5^, none of the other σ^EcfG^ proteins is regulated by NepR or one of its putative homologues, consistent with the finding that σ^EcfG1^ is the only major sigma factor responsible for GSR induction upon stress.

### Transcriptional regulation of *ecfG* genes and control of protein levels

**Fig 5:**
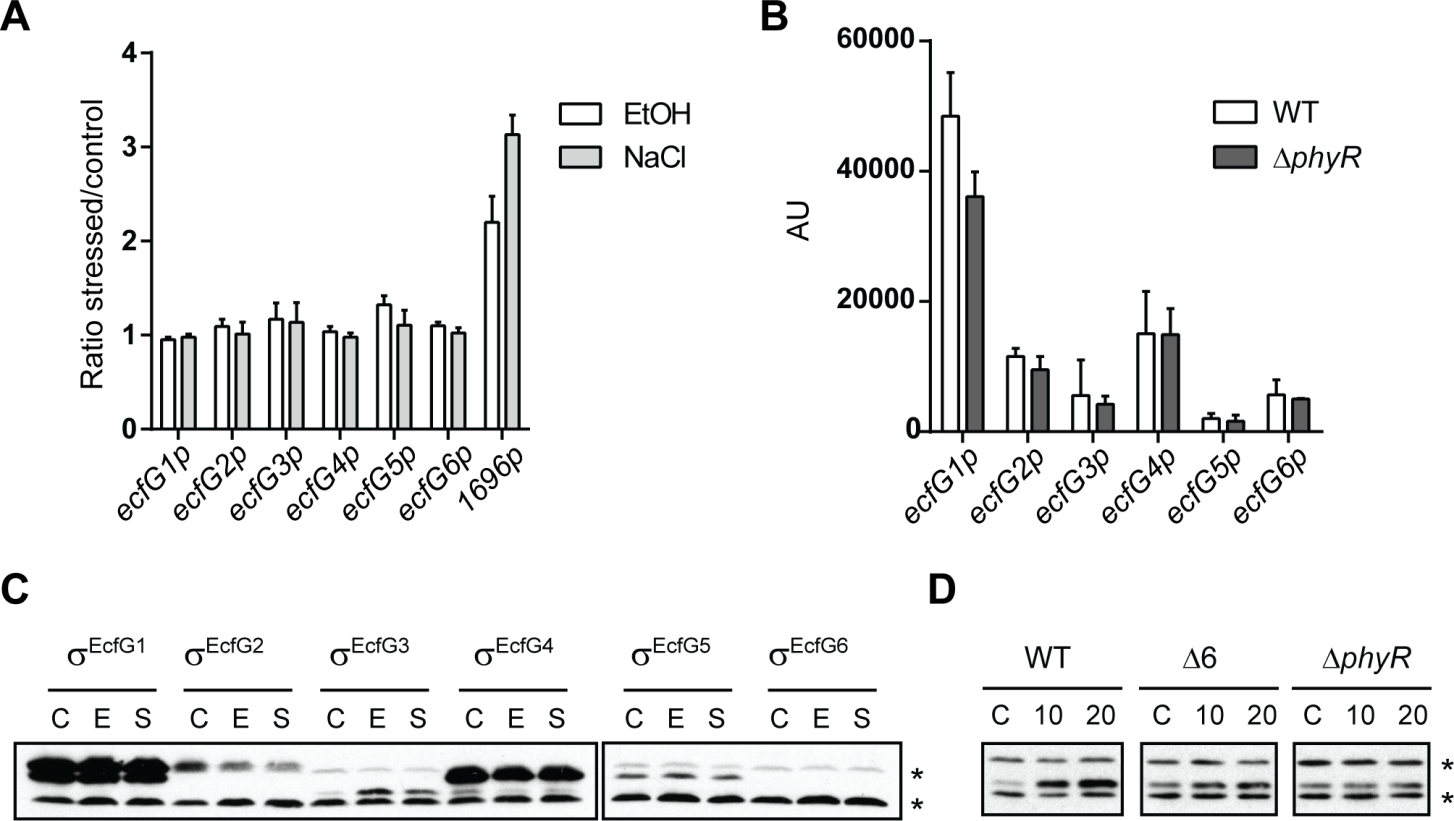
Regulation of *ecfG* genes and σ^EcfG^ proteins. A. Transcriptional regulation of *ecfG* genes in response to stress. Luciferase activity of *luxCDABE* transcriptional fusions to *ecfG* promoters in the wild-type strain exposed to ethanol (white bars) or salts (light grey bars). The ratios of luciferase activity of stressed and unstressed cultures 50 min after stress exposure are shown. The response of the promoter of MexAM1_META1p1696 is shown as an example of a verified GSR target (see main text). B. Dependency of *ecfG* promoters on PhyR. Luciferase activity of *luxCDABE* transcriptional fusions to *ecfG* promoters in the wild-type strain and in the Δ*phyR* mutant in absence of stress. AU, arbitrary units. C and D. Regulation of σ^EcfG^ protein levels. C. Single *ecfG* mutants were complemented with the respective σ^EcfG^ version carrying a C-terminal HA tag. The strains were exposed to water (control, C), ethanol (E) or salt (S) for 20 min, before the samples were analyzed by Western blot using anti-HA antibodies. D. Protein levels of the σ^EcfG3^-HA version were followed in the Δ*ecfG3*, Δ*phyR* or Δ6 mutants upon ethanol exposure for 10 and 20 minutes or without stress (control, C). Asterisks indicate unspecific bands detected in all samples.

Since the activity of most σ^EcfG^ proteins did not seem to be controlled through binding of NepR or any of its homologues, other possible levels of regulation were analyzed. We first tested whether σ^EcfG^ proteins were regulated at the transcriptional level. Although none of the genes possesses a putative σ^EcfG^-type promoter, it is in principle possible that they are regulated by a transcription factor itself controlled by one of the σ^EcfG^ sigma factors, since the regulon comprises several transcriptional regulators (S2 Table). To analyze this possibility, promoter activities of *ecfG* genes upon stress exposure were measured using transcriptional promoter fusions to *luxCDABE* in the wild-type background. Ethanol, salt or sucrose was added to the cultures and luciferase activity was followed. None of the *ecfG* promoters was induced upon stress exposure (Fig 5A), and basal levels of all promoters were identical in the wild type and the *phyR* mutant (Fig 5B), suggesting that none of the *ecfG* genes are regulated at the transcriptional level.

We next checked protein levels. Each *ecfG* ORF was cloned in pCM62HA to generate a C-terminally HA-tagged σ^EcfG^ protein. HA-tagged σ^EcfG1^ and σ^EcfG2^ could complement the sextuple mutant, suggesting functionality of the fusion proteins (S4A Fig). Each plasmid was introduced in the corresponding single mutant, and protein levels upon salts or ethanol exposure were followed by WB using anti-HA antibodies (Fig 5C). All σ^EcfG^ proteins except σ^EcfG6^ could be detected. σ^EcfG1^, σ^EcfG4^ and σ^EcfG5^ showed no difference in protein levels following stress exposure. σ^EcfG2^ seemed to decrease after stress exposure, whereas σ^EcfG3^ accumulated after cells were exposed to salt or ethanol. To analyze whether the increase in σ^EcfG3^ protein levels is dependent on the PhyR cascade, the same experiments were repeated in the *phyR* or sextuple *ecfG* background. As shown in Fig 5D, no difference in σ^EcfG3^ levels after exposure to ethanol could be detected in these backgrounds. Thus, σ^EcfG3^ protein levels are increased upon salt or ethanol exposure in a PhyR/σ^EcfG^-dependent manner.

Altogether, these results indicate that σ^EcfG^ proteins show different levels of regulation: σ^EcfG1^ and σ^EcfG5^ are controlled by binding to NepR, presumably altering their activities, whereas for σ^EcfG3^ it is protein abundance that is regulated upon stress exposure in a PhyR cascade-dependent manner.

### Two NepR homologues bind PhyR

**Fig 6:**
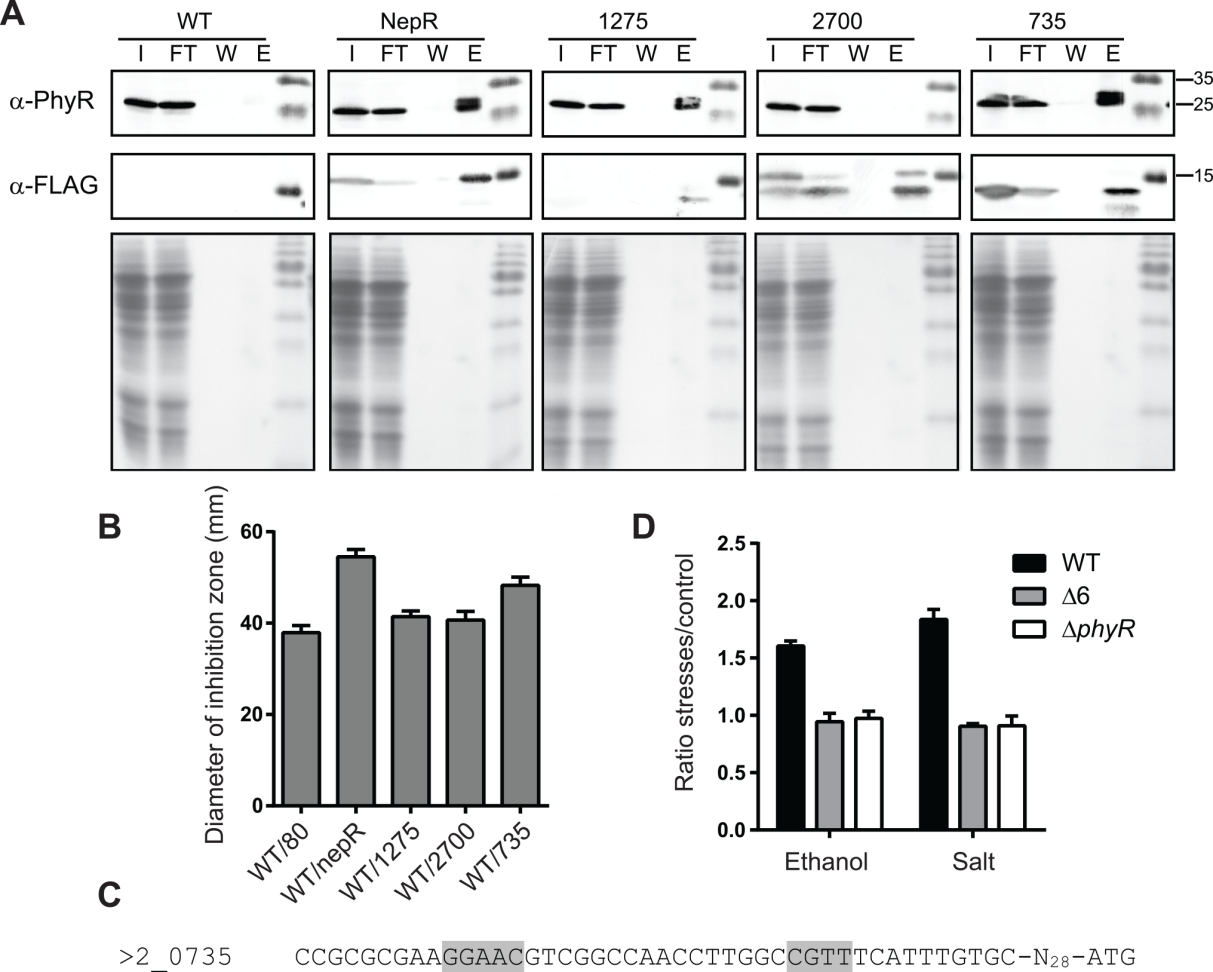
Characterization of NepR paralogues. A. Interactions between NepR paralogues and PhyR. Co-immunoprecipitation of C-terminal triple FLAG-tagged NepR paralogues. The control strain (WT) bears the empty plasmid pCM80. The input (I), flow-through (FT), last washing step (W) and elution (E) fractions were analyzed by Western blot using anti-PhyR (upper panel) or anti-FLAG (middle panel) antibodies, or stained with Coomassie Blue (lower panel). B. Stress sensitivity of strains overexpressing *nepR* paralogues. Methylglyoxal sensitivity of the wild-type strains overexpressing *nepR* (WT/nepR) or one of the putative *nepR* homologues (WT/1275, WT/2700, WT/735) from the *mxaF* promoter, or bearing the empty plasmid (WT/80). Data are displayed as means +/- SD of three independent biological replicates. C. Putative σ^EcfG^-dependent promoter of META2_0735. The -35 and -10 boxes are highlighted in grey. D. Activity of the *735p*::*luxCDABE* transcriptional fusion in response to ethanol or salt in the wild-type, Δ6 or Δ*phyR* strain. Values are given as means +/- SD of two independent biological replicates.

Because no interactions between the putative NepR homologues and the σ^EcfG^ proteins could be observed, we wondered whether they were involved in the PhyR cascade by other means. Most NepR residues known to interact with PhyR based on *C*. *crescentus* and *S*. *melonis* PhyR-NepR complexes are conserved in these proteins (S2C Fig), suggesting they can bind PhyR, and for two of them (MexAM1_META1_2700 and MexAM1_META2_0735), putative σ^EcfG^-type promoters are found in the upstream region of their encoding genes, linking them to the GSR. Interactions between PhyR and NepR homologues were analyzed using co-immunoprecipitation experiments using C-terminal triple FLAG-tagged versions of NepR or NepR paralogues. The NepR and MexAM1_META2p0735 triple FLAG-tagged versions were functional as judged by methylglyoxal sensitivity assays (S4B Fig and see below for comparison with the untagged versions). As shown in Fig 6A, PhyR could be co-immunoprecipitated with NepR, MexAM1_META1p1275 and MexAM1_META2p0735; no co-immunoprecipitation of PhyR was observed for MexAM1_META1p2700 or for a control strain bearing the empty pCM80 plasmid. Altogether, these data indicate that in addition to NepR, PhyR interacts with MexAM1_META1p1275 and MexAM1_META2p0735. However, our data suggest that these proteins do not act as anti-sigma factor since they were not found to interact with any σ^EcfG^ protein, although it is possible that interactions were missed in our analysis. In case absence of interaction was true, NepR homologues might then rather act as anti-anti-anti-sigma factors.

We thus next sought to analyze whether the NepR homologues were negative regulator of the PhyR cascade. Each gene was expressed from the strong *mxaF* promoter in the wild type and stress sensitivity of the resulting strains tested. As shown in Fig 6B, overexpression of *nepR* or *mexAM1_meta2p0735* rendered the strain more sensitive to methylglyoxal than the wild-type bearing the empty plasmid, whereas no difference was observed for *mexAM1_meta1p1275* and *mexAM1_meta1p2700*. These data are in agreement with a role of MexAM1_META2p0735 as a negative regulator of PhyR-dependent response. Since the gene possesses a putative σ^EcfG^-type promoter (Fig 6C), we also checked whether its expression was induced upon stress exposure. As shown in Fig 6D, the activity of the promoter was increased after treatment with ethanol or salt, and this induction was abolished in a Δ6 or Δ*phyR* background.

### Conservation of σ^EcfG^ proteins in *Methylobacterium* species

**Fig 7:**
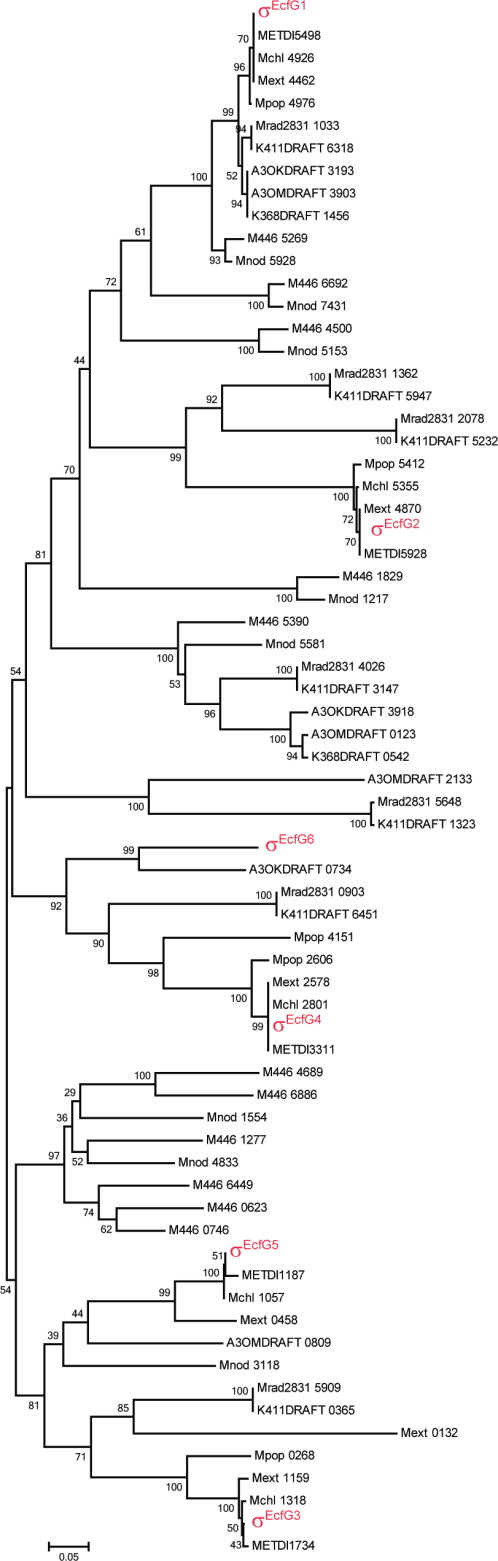
Evolutionary relationships of σ^EcfG^ proteins of *Methylobacterium* species. The evolutionary history was inferred using the Neighbor-Joining method [26]. The percentage of replicate trees in which the associated taxa clustered together in the bootstrap test (500 replicates) are shown next to the branches [27]. The tree is drawn to scale, with branch lengths in the same units as those of the evolutionary distances used to infer the phylogenetic tree. The evolutionary distances were computed using the Poisson correction method [28] and are in the units of the number of amino acid substitutions per site. The analysis involved 69 amino acid sequences. All positions with less than 95% site coverage were eliminated. That is, fewer than 5% alignment gaps, missing data, and ambiguous bases were allowed at any position. There were a total of 171 positions in the final dataset.

Having analyzed the topology of the core cascade in *M*. *extorquens* AM1, we wondered whether σ^EcfG^ proteins were conserved in other *Methylobacterium* species. A search for ECF sigma factors in *Methylobacterium* species indicated they commonly harbor several *ecfG* genes (see S1 and S3 Tables). Phylogenetic analysis of σ^EcfG^ proteins of Methylobacterium species suggested that only σ^EcfG1^ is conserved in all *Methylobacterium* strains considered (Fig 7). The other σ^EcfG^ proteins were only found in very closely related species, such as *M*. *extorquens* DM4, PA1 and CM4, and *M*. *nodulans* (Fig 7). Strains of more distantly related *Methylobacterium* species have their own set of σ^EcfG^ proteins, the number of which varies (Fig 7). Thus, in addition to σ^EcfG1^, which is found in all species in agreement with its central role in the partner switch, *Methylobacterium* species possess different sets of σ^EcfG^ proteins, illustrating the plasticity of the system controlling the GSR in this alphaproteobacterial genus.

## Discussion

**Fig 8:**
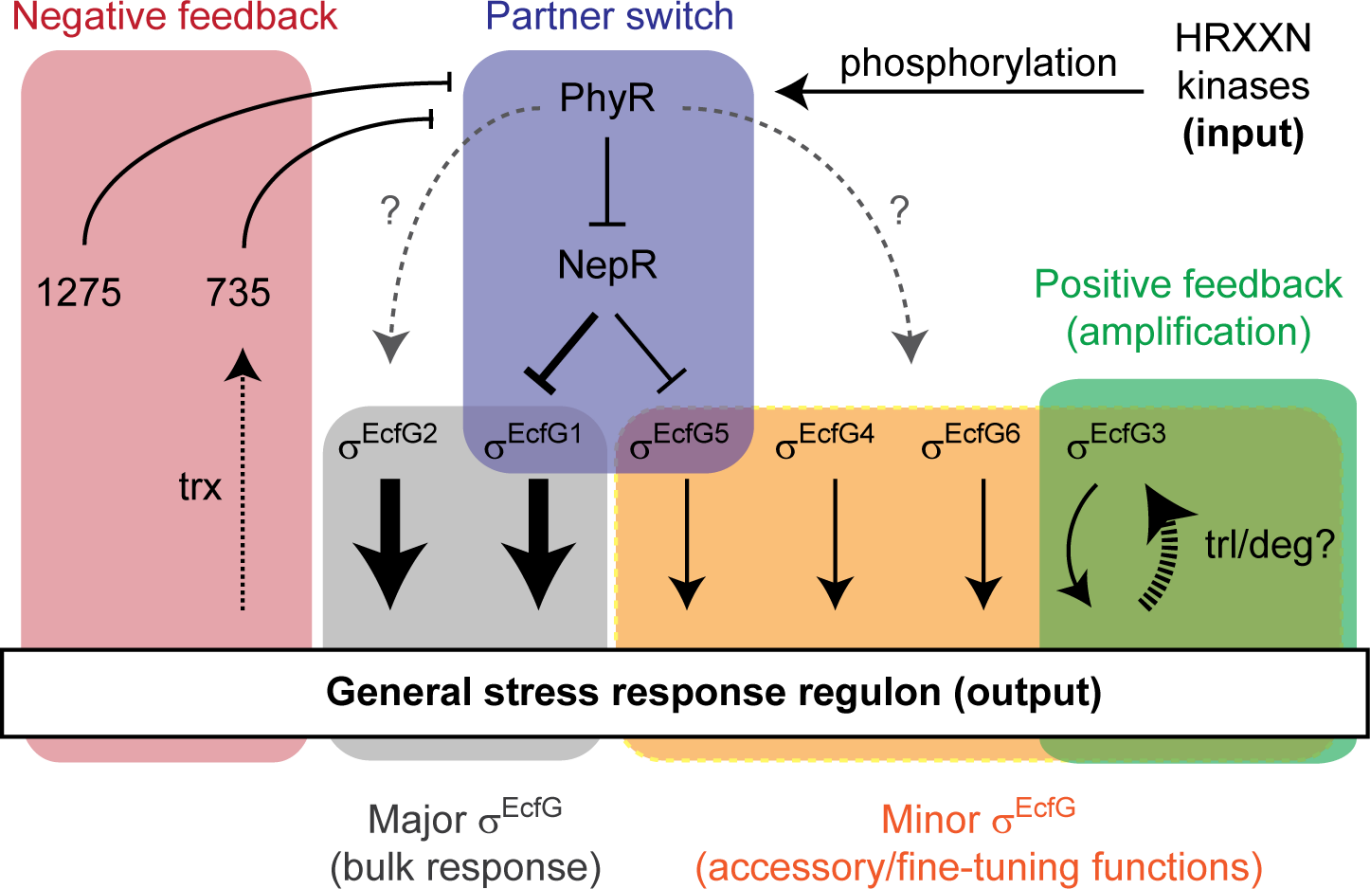
Model of the GSR in *M*. *extorquens* AM1. Activating and repressing relationships are indicted by solid black arrows and bars, respectively. The grey dotted arrow with question mark indicates an unknown, likely indirect positive effect of PhyR on σ^EcfG^ proteins. The dotted black arrow with trx (“transcription”) indicates transcriptional regulation of MexAM1_META2p0735 by the GSR. The dashed arrow with trl/deg (“translation/degradation”) indicates an increase of σ^EcfG3^ protein levels upon stress treatment in a GSR-dependent manner. Individual modules are highlighted by colored boxes.

*M*. *extorquens* AM1 possesses a rather complex PhyR-NepR-σ^EcfG^ cascade compared to other Alphaproteobacteria. With this study, we provide an advanced picture of the regulatory network underlying the GSR in this organism, schematized in Fig 8. Our study identifies two σ^EcfG^ sigma factors, σ^EcfG1^ and σ^EcfG2^, as having a major contribution to the regulation of the GSR, whereas up to four of the remaining σ^EcfG^ proteins are also involved in the response, but to a lesser extent. Our data suggest that these sigma factors are regulated through different mechanisms at the posttranscriptional level: sequestration by the anti-sigma factor NepR for σ^EcfG1^ and σ^EcfG5^, and regulation of protein levels upon stress exposure in a *phyR* and *ecfG* dependent manner for σ^EcfG3^. We also characterized two putative NepR homologues as potential negative regulators of the response, interacting with PhyR but not with any σ^EcfG^-type sigma factor, one of which is itself a target of the GSR. Thus, our study suggests that σ^EcfG^ sigma factors and anti-sigma factors of *M*. *extorquens* are not fully redundant in the control of the GSR.

Our study indicates that *Methylobacterium* σ^EcfG^ paralogues are embedded into the GSR regulatory network in a different way from what was described in other Alphaproteobacteria. In *C*. *crescentus* and *S*. *melonis*, *ecfG2* transcription is controlled by σ^EcfG1^, and σ^EcfG2^ is not regulated through NepR binding [9, 10]. In *R*. *etli*, the two σ^EcfG^ proteins have been proposed to act in parallel, controlling distinct sets of genes and being responsible for resistance to different stresses, i.e. heat shock and oxidative stress, but whether they are controlled by NepR is not known [15, 38]. In *M*. *extorquens*, there is no σ^EcfG^ that controls the activity of all other σ^EcfG^ protein(s), in contrast to *Caulobacter* and *Sphingomonas*, and apparently no stress-specific phenotype exists for any of the single *ecfG* mutants, suggesting that the situation is also different from the one in *R*. *etli*. Instead, *M*. *extorquens* combines control by NepR (for σ^EcfG1^ and σ^EcfG5^), PhyR/σ^EcfG^-dependent regulation at the posttranscriptional level (for σ^EcfG3^), and an apparently constitutively active σ^EcfG^ (σ^EcfG2^). Hence, the regulatory cascades controlling the GSR seem to function in different ways in Alphaproteobacteria, with σ^EcfG^ paralogues playing distinct roles. This is in agreement with phylogenetic analyses which indicate that σ^EcfG^ paralogues of different species do not cluster together in the phylogenetic tree, suggesting they arose from different duplication events ([15] and S1 Fig). Despite these differences, some regulatory features of the response seem to be conserved.

In the current model of the alphaproteobacterial GSR in non-*Methylobacterium* systems, positive autoregulation of the components of the partner switch in response to acute stress is expected to provide higher levels of σ^EcfG^, NepR and PhyR after activation compared to uninduced condition [1]. Such positive feedback allows obtaining high levels of the sigma factor in maximum-stress condition while keeping low levels in pre-stress condition, increasing the capacity of regulation [39]; co-regulation of the three proteins also probably allows maintaining a stoichiometry necessary for full responsiveness of the partner switch. In *M*. *extorquens* AM1, in contrast, σ^EcfG1^ is apparently not autoregulated, whereas σ^EcfG^-dependent promoters were mapped or predicted upstream of *phyR* and *nepR*, respectively, suggesting that these components are subjected to autoregulation. In addition, the second main σ^EcfG^ of the system, σ^EcfG2^, is apparently not regulated by NepR nor activated upon GSR induction, and thus seems to represent a constitutively active σ^EcfG^. A positive feedback loop might be provided by σ^EcfG3^, which is activated upon stress exposure in a σ^EcfG^- and PhyR-dependent manner. The regulation occurs at the protein level, but not at the transcriptional level, suggesting regulation by proteolysis or at the level of translation, possibly by a small RNA. Interestingly, although the level of control is different, the principle of regulation corresponds to the situation observed in *C*. *crescentus* and *S*. *melonis*, where *ecfG2* is regulated at the transcriptional level by the main, NepR-controlled σ^EcfG1^. Such a motif (type I coherent feed forward loop) is proposed to provide a way to filter transient signals and to prolong the response after the system is turned off [40]. It thus seems plausible that convergent evolution in different Alphaproteobacteria led to similar principles of regulation with distinct mechanisms. Additionally, it is also possible that the regulation of σ^EcfG3^ integrates other signals into the system to regulate σ^EcfG3^-dependent GSR targets in response to PhyR-NepR-σ^EcfG^ and σ^EcfG3^-activating stimuli. Although PhyR-dependent regulatory mechanisms were identified for σ^EcfG1^, σ^EcfG3^ and σ^EcfG5^, it is worth noting that a mutant deleted for these three genes does not show stress sensitivity, whereas a *phyR* mutant does, suggesting that the remaining σ^EcfG^ proteins, notably σ^EcfG2^, should be controlled by PhyR, by a mechanism which still needs to be identified.

Another common feature in regulatory systems is the presence of negative feedback loops that allow, for example, to mount faster responses [39]. Here, we identified two NepR homologues for which our data suggest they bind PhyR but not any σ^EcfG^ sigma factors. Since one of the genes harbors a σ^EcfG^-dependent promoter, and is upregulated upon stress exposure in a σ^EcfG^/PhyR-dependent manner, the role of this NepR homologue could be to provide a negative feedback in the cascade, by competing with NepR for binding to PhyR, releasing NepR to bind σ^EcfG1^ and σ^EcfG5^ and thus limiting GSR activation. In agreement with such a role, overexpression of this gene leads to downregulation of the GSR. However, more experiments are needed to establish such a role, notably the measurement of PhyR affinities for NepR and its homologues as well as their relative concentration in cells. Except in *M*. *extorquens*, NepR paralogues have only been studied in *S*. *meliloti*, but there both NepR proteins (RsiA1 and RsiA2) bind σ^EcfG^ (RpoE2) and the two PhyR proteins (RsiB1 and RsiB2) [13], suggesting different functions compared to the NepR paralogues of *M*. *extorquens* AM1. In general, little is known concerning possible negative feedbacks in the alphaproteobacterial GSR. In *S*. *melonis*, a PhyR phosphatase-encoding gene is expressed from a σ^EcfG^-dependent promoter and it has been shown that lack of this protein leads to high (lethal) GSR activity levels [10, 41]. However, it is currently not known whether its expression is induced upon stress exposure, and whether its activity is regulated by additional signals. Possibly, in *M*. *extorquens*, NepR homologues might show other levels of regulation, such as proteolysis, in addition to transcriptional control by σ^EcfG^.

In conclusion, our study suggests that despite its complex layout and the apparent lack of many conserved features, the GSR of *M*. *extorquens* has nevertheless maintained the capacity to launch an efficient response, which integrates positive and negative feedback in this regulatory network. In contrast to other Alphaproteobacteria, *M*. *extorquens* seems to employ a plethora of different mechanisms to achieve this, but it is not clear at the moment whether this regulatory layout confers any advantages. While we have only focused on the core elements of the partner switch–PhyR, NepR and σ^EcfG^–in the current study, it is noteworthy that *M*. *extorquens* harbors the largest set of HRXXN kinases of any Alphaproteobacteria known to date, all of which potentially influence PhyR phosphorylation. Hence, the GSR in *M*. *extorquens* is an attractive model to analyze the role of paralogous proteins in complex regulatory systems.

## Supporting Information

S1 Fig Evolutionary relationships of σ^EcfG^ proteins in selected members of the Order Rhizobiales. Sequences were aligned with ClustalW with MEGA6 default parameters. The evolutionary history was inferred using the Neighbor-Joining method [26]. The percentage of replicate trees in which associated taxa clustered together in the bootstrap test (500 replicates) are shown next to the branches [27]. The tree is drawn to scale, with branch lengths in the same units as those of the evolutionary distances used to infer the phylogenetic tree. The evolutionary distances were computed using the Poisson correct ion method [28] and are in the units of the number of amino acid substitutions per site. The analysis involved 33 amino acid sequences. All positions with less than 95% site coverage were eliminated. That is, fewer than 5% alignment gaps, missing data, and ambiguous residues were allowed at any position. There were a total of 150 positions in the final dataset. Evolutionary analyses were conducted in MEGA6 [24].
(EPS)

S2 Fig Alignment of σ^EcfG^ and NepR homologues of *M*. *extorquens* AM1. The sequences were aligned with ClustalW [25]. Identical or similar residues conserved in at least 70% of the sequences are highlighted. A. Alignment of σ^EcfG^ proteins. Grey bars indicate regions σ_2.4_ and σ_4.2_ involved in -10 and -35 boxes binding, respectively. B. Alignment of NepR, putative NepR homologues and the N-terminal part of σ^EcfG^ proteins. C. Alignment of NepR with putative NepR homologues. A and C. Dots indicate positions corresponding to amino acid involved in interactions with NepR or σ^EcfG^ based on results from homology modelling of the σ^EcfG^-NepR complex of *S*. *melonis* Fr1 modeled on the structure of the PhyR_SL_-NepR complex of *S*. *melonis* Fr1 [37], from mutagenesis studies and bacterial two-hybrid experiments with *S*. *melonis* Fr1 proteins [37] or pull-down experiments with *C*. *crescentus* proteins [42] testing for interactions. Color coding is as follows: black, residue not tested but involved in the interaction with NepR based on homology modelling; orange, alanine substitution results in moderate defect in interaction with PhyR and no or only with defect with σ^EcfG^; red, alanine substitution results in severe defect in interaction.
(EPS)

S3 Fig Analysis of σ^EcfG1^ isoforms. A. Nucleotide sequence corresponding to the end of the 5'UTR and the beginning of *ecfG1* ORF of the wild-type (FL) and the two mutated alleles (v1 and v2). The start codons, or mutated start codons in the v1 and v2 alleles, are shown in bold letters. The N-terminal amino acid sequences of the expected proteins are indicated below the nucleotide sequence. B. Analysis of σ^EcfG1^ isoforms by Western Blot using anti-σ^EcfG1^antibodies. The strains are indicated as follows: FL, Δ6/pCM62_ecfG1; v1, Δ6/pCM62_ecfG1v1; v2, Δ6/pCM62_ecfG1v2. C. Methylglyoxal sensitivity of strains expressing σ^EcfG1^ or only one of its isoform. Strains are indicated as follows: wt/pCM62, Δ6/pCM62, Δ6/pCM62_ecfG1 (Δ6/FL), Δ6/pCM62_ecfG1v1 (Δ6/v1) and Δ6/pCM62_ecfG1v2 (Δ6/v2).
(TIF)

S4 Fig Activity of the HA-tagged σ^EcfG^ proteins and triple flag-tagged NepR. A. Methylglyoxal sensitivity of the sextuple mutant expressing each HA-tagged σ^EcfG^. The wild-type strain and the sextuple mutant containing the empty pCM62HA plasmid are shown as control. B. Methylglyoxal sensitivity of the wild-type strain overexpressing triple flag-tagged versions of NepR or NepR paralogues. The wild-type strain and the sextuple mutant containing the empty pCM80 are shown as control.
(TIF)

S1 Table σ^EcfG^ in Alphaproteobacteria. (DOCX)

S2 Table Microarray data. (XLSX)

S3 Table σ^EcfG^ proteins of *Methylobacterium* species. (DOCX)

S4 Table Strains and plasmids. (DOCX)

S5 Table Primers. (DOCX)
