# Supplementary figures and images for: Multiple σEcfG and NepR Proteins Are Involved in the General Stress Response in Methylobacterium extorquens
Source: PLoS One. 2016 Mar 30;11(3):e0152519. doi: 10.1371/journal.pone.0152519 (PMC4814048; doi:10.1371/journal.pone.0152519)

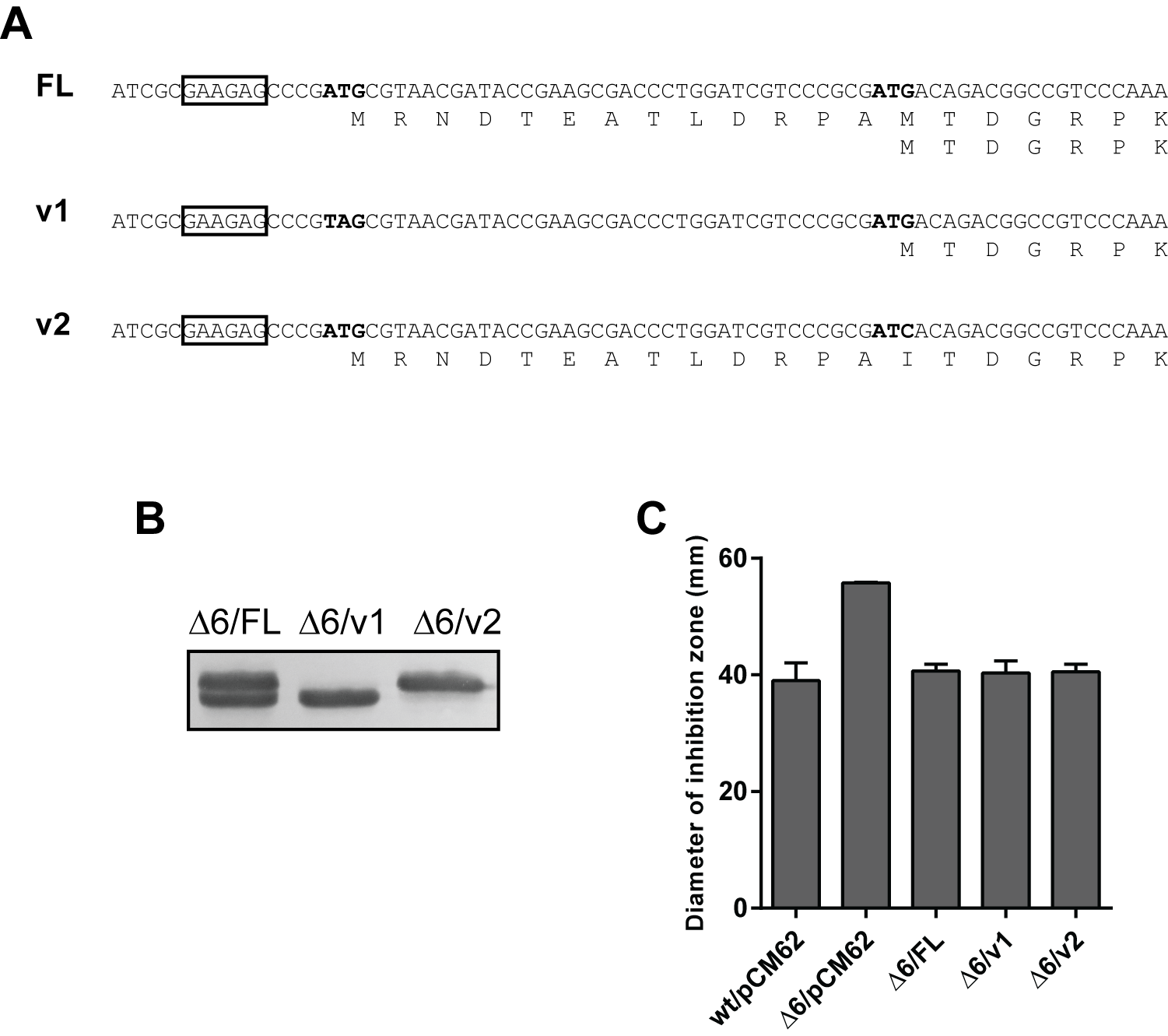

Supplement: S3 Fig — A. Nucleotide sequence corresponding to the end of the 5'UTR and the beginning of ecfG1 ORF of the wild-type (FL) and the two mutated alleles (v1 and v2). The start codons, or mutated start codons in the v1 and v2 alleles, are shown in bold letters. The N-terminal amino acid sequences of the expected proteins are indicated below the nucleotide sequence. B. Analysis of σEcfG1 isoforms by Western Blot using anti-σEcfG1antibodies. The strains are indicated as follows: FL, Δ6/pCM62_ecfG1; v1, Δ6/pCM62_ecfG1v1; v2, Δ6/pCM62_ecfG1v2. C. Methylglyoxal sensitivity of strains expressing σEcfG1 or only one of its isoform. Strains are indicated as follows: wt/pCM62, Δ6/pCM62, Δ6/pCM62_ecfG1 (Δ6/FL), Δ6/pCM62_ecfG1v1 (Δ6/v1) and Δ6/pCM62_ecfG1v2 (Δ6/v2). (TIF) [file pone.0152519.s003.tif]

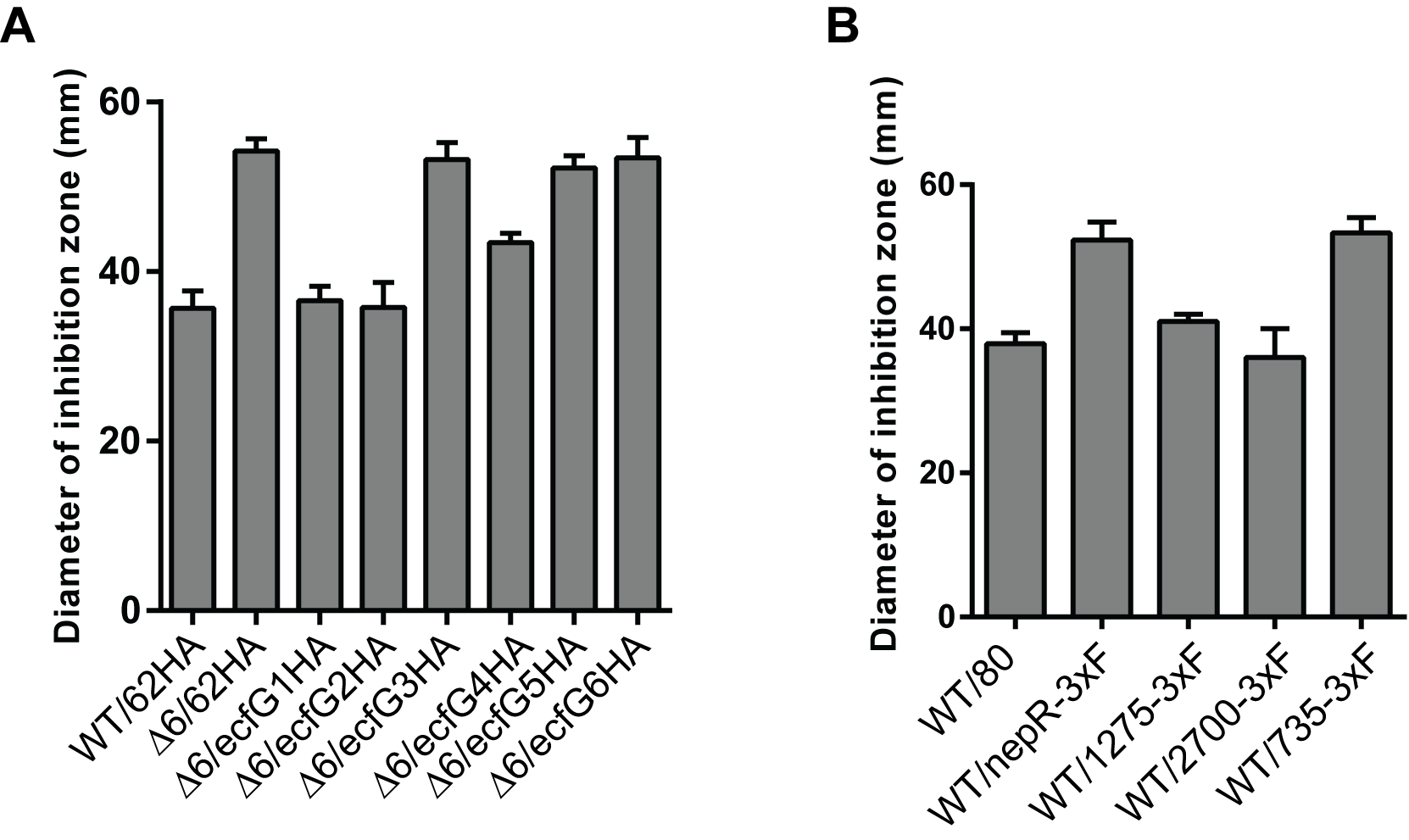

Supplement: S4 Fig — A. Methylglyoxal sensitivity of the sextuple mutant expressing each HA-tagged σEcfG. The wild-type strain and the sextuple mutant containing the empty pCM62HA plasmid are shown as control. B. Methylglyoxal sensitivity of the wild-type strain overexpressing triple flag-tagged versions of NepR or NepR paralogues. The wild-type strain and the sextuple mutant containing the empty pCM80 are shown as control. (TIF) [file pone.0152519.s004.tif]
