# Supplementary material for: Multiple σEcfG and NepR Proteins Are Involved in the General Stress Response in Methylobacterium extorquens
Source: PLoS One. 2016 Mar 30;11(3):e0152519. doi: 10.1371/journal.pone.0152519 (PMC4814048; doi:10.1371/journal.pone.0152519)
Supplement: S1 Table — (DOCX) [file pone.0152519.s005.docx]

**S1 Table. σ^EcfG^ in Alphaproteobacteria.**

| **Strain^a^** | **Number of *ecfG* genes^b^** | ***ecfG* at *phyR* locus^c^** | **Additional *ecfG* genes** | **Family** | **Order** |
| --- | --- | --- | --- | --- | --- |
| Asticcacaulis excentricus CB 48 | 0 |  |  | Caulobacteraceae | Caulobacterales |
| Brevundimonas subvibrioides ATCC 15264 | 1 | Bresu_0163 |  | Caulobacteraceae | Caulobacterales |
| Caulobacter crescentus CB15 | 2 | CC_3475 | CC2883 | Caulobacteraceae | Caulobacterales |
| Caulobacter segnis | 1 | Cseg_4035 |  | Caulobacteraceae | Caulobacterales |
| Caulobacter sp. K31 | 4 | Caul_0091 | Caul_1961, Caul_2060, Caul_2683 | Caulobacteraceae | Caulobacterales |
| Phenylobacterium zucineum HLK1 | 2 | PHZ_c0301 | PHZ_c1907 | Caulobacteraceae | Caulobacterales |
| Magnetococcus marinus | 0 |  |  | Magnetococcaceae | Magnetococcales |
| Parvularcula bermudensis HTCC2503 | 1 | PB2503_10164 |  | Parvularculaceae | Parvularculales |
| Bartonella australis Aust/NH1 | 1 | BAnh1_10730 |  | Bartonellaceae | Rhizobiales |
| Beijerinckia indica subsp. indica ATCC 9039 | 1 | Bind_3241 |  | Beijerinckiaceae | Rhizobiales |
| Methylocella silvestris BL2 | 1 | Msil_0284 |  | Beijerinckiaceae | Rhizobiales |
| Bradyrhizobium japonicum USDA 110 | 1 | blr7797 |  | Bradyrhizobiaceae | Rhizobiales |
| Nitrobacter hamburgensis X14 | 1 | Nham_3572 |  | Bradyrhizobiaceae | Rhizobiales |
| Oligotropha carboxidovorans OM5 | 1 | OCA5_c07210 |  | Bradyrhizobiaceae | Rhizobiales |
| Rhodopseudomonas palustris TIE-1 | 1 | Rpal_4706 |  | Bradyrhizobiaceae | Rhizobiales |
| Brucella melitensis M5-90 | 1 | BM590_A1648 |  | Brucellaceae | Rhizobiales |
| Ochrobactrum anthropi ATCC 49188 | 3 | Oant_1248 | Oant_4302, Oant_4309 | Brucellaceae | Rhizobiales |
| Hyphomicrobium denitrificans 1NES1^d^ | 2 | HYPDE_29698  HYPDE_37078 |  | Hyphomicrobiaceae | Rhizobiales |
| Hyphomicrobium denitrificans ATCC 51888^d^ | 2 | Hden_2688 Hden_1366 |  | Hyphomicrobiaceae | Rhizobiales |
| Hyphomicrobium nitrativorans NL23 | 1 | W911_07960 |  | Hyphomicrobiaceae | Rhizobiales |
| Hyphomicrobium sp. MC1 | 3 | HYPMC_0698 | HYPMC_2622, HYPMC_3285 | Hyphomicrobiaceae | Rhizobiales |
| Pelagibacterium halotolerans B2 | 1 | KKY_2053 |  | Hyphomicrobiaceae | Rhizobiales |
| Rhodomicrobium vannielii ATCC 17100 | 1 | Rvan_2509 |  | Hyphomicrobiaceae | Rhizobiales |
| Methylobacterium extorquens AM1 | 6 |  | META1_4906, META1_5327, META1_0932, META1_2698, META2_0154, META2_1029 | Methylobacteriaceae | Rhizobiales |
| Methylobacterium extorquens CM4 | 5 |  | Mchl_4926 , Mchl_5355 , MChl_1318, Mchl_2801 , Mchl_1057 | Methylobacteriaceae | Rhizobiales |
| Methylobacterium extorquens DM4 | 5 |  | METDI_5498 , METDI_5928 , METDI1734 , METDI3311 , METDI_1187 | Methylobacteriaceae | Rhizobiales |
| Methylobacterium extorquens PA1 |  |  | Mext_4462 , Mext_4870 , Mext_1159, Mext_2578 , Mext_0458, Mext_0132^e^ | Methylobacteriaceae | Rhizobiales |
| Methylobacterium nodulans ORS2060 | 8 |  | Mnod_5928, Mnod_5581, Mnod_7431 , Mnod_5153 , Mnod_1217 , Mnod_4833, Mnod_1554, Mnod_3118 | Methylobacteriaceae | Rhizobiales |
| Methylobacterium populi BJ001 | 5 |  | Mpop_4976 , Mpop_5412, Mpop_0268 , Mpop_2606 , Mpop_4151 | Methylobacteriaceae | Rhizobiales |
| Methylobacterium radiotolerans JCM2831 | 7 |  | Mrad2831_1033 , Mrad2831_5909, Mrad2831_1362 , Mrad2831_2078 , Mrad2831_0903 , Mrad2831_4026 , Mrad2831_5648 | Methylobacteriaceae | Rhizobiales |
| Methylobacterium sp. 4 46 | 11 |  | M446_5269, M446_5390 , M446_6692 , M446_4500, M446_1829, M446_0746 , M446_1277 , M446_0623 , M446_6449 , M446_6886, M446_4689 | Methylobacteriaceae | Rhizobiales |
| Methylocystis sp. SC2 | 2 | BN69_1929  BN69_2208 |  | Methylocsytaceae | Rhizobiales |
| Chelativorans sp. BNC1 | 2 | Meso_3107 | Meso_1447 | Phyllobacteriaceae | Rhizobiales |
| Mesorhizobium australicum WSM2073 | 2 | Mesau_01468 | Mesau_05485 | Phyllobacteriaceae | Rhizobiales |
| Mesorhizobium loti MAFF303099 | 1 | mll3697 |  | Phyllobacteriaceae | Rhizobiales |
| Agrobacterium fabrum str. C58 | 3 | Atu4160 | Atu5283, Atu5490 | Rhizobiaceae | Rhizobiales |
| Agrobacterium radiobacter K84 | 1 | Arad_3433 |  | Rhizobiaceae | Rhizobiales |
| Agrobacterium vitis S4 | 2 | Avi_5597 | Avi_5992 | Rhizobiaceae | Rhizobiales |
| Liberibacter crescens BT-1 | 0 |  |  | Rhizobiaceae | Rhizobiales |
| Rhizobium etli CFN 42 | 2 | RHE_CH03273 | RHE_PF00052 | Rhizobiaceae | Rhizobiales |
| Rhizobium leguminosarum bv. trifolii WSM1325 | 4 | Rleg_3276 (chr) | Rleg_6866 (plas), Rleg_5591 (plas), Rleg_5820 (plas) | Rhizobiaceae | Rhizobiales |
| Rhizobium tropici CIAT 899 | 3 | RTCIAT899_CH13715 | RTCIAT899_CH11945, RTCIAT899_PC09455 | Rhizobiaceae | Rhizobiales |
| Sinorhizobium meliloti 1021 | 2 | SMc01506 | SM_b21484 | Rhizobiaceae | Rhizobiales |
| Parvibaculum lavamentivorans DS-1 | 1 | Plav_0053 |  | Rhodobiaceae | Rhizobiales |
| Azorhizobium caulinodans ORS 571 | 1 | AZC_4180 |  | xanthobacteraceae | Rhizobiales |
| Starkeya novella DSM 506 | 1 | Snov_0053 |  | xanthobacteraceae | Rhizobiales |
| Xanthobacter autotrophicus Py2 | 1 | Xaut_2021 |  | xanthobacteraceae | Rhizobiales |
| Hirschia baltica ATCC 49814 | 1 | Hbal_2034 |  | Hyphomonadaceae | Rhodobacterales |
| Hyphomonas neptunium ATCC 15444 | 2 | HNE_2340 | HNE_0281 | Hyphomonadaceae | Rhodobacterales |
| Maricaulis maris MCS10 | 0 |  |  | Hyphomonadaceae | Rhodobacterales |
| Dinoroseobacter shibae DFL 12 | 1 | Dshi_3835 |  | Rhodobacteraceae | Rhodobacterales |
| Jannaschia sp. CCS1 | 1 | Jann_3565 |  | Rhodobacteraceae | Rhodobacterales |
| Ketogulonicigenium vulgare WSH-001 | 5 | KVU_PB0108 | KVU_PA0105, KVU_0755, KVU_0773, KVU_1422 | Rhodobacteraceae | Rhodobacterales |
| Leisingera methylohalidivorans DSM 14336 | 2 | METH_19420 | METH_19450 | Rhodobacteraceae | Rhodobacterales |
| Paracoccus aminophilus JCM 7686 | 1 | JCM7686_3466 |  | Rhodobacteraceae | Rhodobacterales |
| Paracoccus denitrificans PD1222 | 2 | Pden_2820 | Pden_1137 | Rhodobacteraceae | Rhodobacterales |
| Phaeobacter gallaeciensis DSM 17395 | 1 | PGA1_c27070 |  | Rhodobacteraceae | Rhodobacterales |
| Rhodobacter capsulatus SB 1003 | 1 | RCAP_rcc02291 |  | Rhodobacteraceae | Rhodobacterales |
| Rhodobacter sphaeroides 2.4.1 | 2 | RSP_2681 | RSP_1272 | Rhodobacteraceae | Rhodobacterales |
| Roseobacter denitrificans OCh 114^d^ | 2 | RD1_0085  RD1_2462 |  | Rhodobacteraceae | Rhodobacterales |
| Roseobacter litoralis Och 149^d^ | 2 | RLO149_c001120  RLO149_p940390 |  | Rhodobacteraceae | Rhodobacterales |
| Ruegeria pomeroyi DSS-3 | 0 |  |  | Rhodobacteraceae | Rhodobacterales |
| Ruegeria sp. TM1040 | 1 | TM1040_2723 |  | Rhodobacteraceae | Rhodobacterales |
| Acetobacter pasteurianus IFO 3283-03 | 2 | APA03_12650 | APA03_12660 | Acetobacteraceae | Rhodospirillales |
| Acidiphilium cryptum JF-5 | 0 |  |  | Acetobacteraceae | Rhodospirillales |
| Gluconacetobacter diazotrophicus PAl 5 | 2 | GDI_1742 | GDI_1743 | Acetobacteraceae | Rhodospirillales |
| Gluconobacter oxydans H24 | 2 | B932_2564 | B932_2565 | Acetobacteraceae | Rhodospirillales |
| Granulibacter bethesdensis CGDNIH1 | 2 | GbCGDNIH1_0754 | GbCGDNIH1_0755 | Acetobacteraceae | Rhodospirillales |
| Azospirillum sp. | 0 |  |  | Rhodospirillaceae | Rhodospirillales |
| Magnetospirillum gryphiswaldense MSR-1 v2 | 0 |  |  | Rhodospirillaceae | Rhodospirillales |
| Rhodospirillum photometricum DSM 122 | 0 |  |  | Rhodospirillaceae | Rhodospirillales |
| Rhodospirillum rubrum ATCC 11170 | 1 | Rru_A3287 |  | Rhodospirillaceae | Rhodospirillales |
| Tistrella mobilis KA081020-065 | 1 | TMO_a0327 |  | Rhodospirillaceae | Rhodospirillales |
| Erythrobacter litoralis HTCC2594 | 1 | ELI_10230 |  | Erythrobacteraceae | Sphingomonadales |
| Novosphingobium aromaticivorans DSM 12444 | 1 | Saro_0485 |  | Sphingomonadaceae | Sphingomonadales |
| Novosphingobium sp. PP1Y | 2 | PP1Y_AT9244 | PP1Y_AT9201 | Sphingomonadaceae | Sphingomonadales |
| Sphingobium chlorophenolicum L-1 | 1 | Sphch_1159 |  | Sphingomonadaceae | Sphingomonadales |
| Sphingomonas sp. MM-1 | 1 | G432_06220 |  | Sphingomonadaceae | Sphingomonadales |
| Sphingopyxis alaskensis RB2256 | 2 | Sala_0286 | Sala_0284 | Sphingomonadaceae | Sphingomonadales |
| Zymomonas mobilis subsp. mobilis ZM4 | 1 | ZMO1404 |  | Sphingomonadaceae | Sphingomonadales |
| Micavibrio aeruginosavorus ARL-13 | 0 |  |  | Top of Form  unclassified Alphaproteobacteria  Bottom of Form | Top of Form  unclassified Alphaproteobacteria  Bottom of Form |
| Polymorphum gilvum SL003B-26A1^f^ | 1 | SL003B_1060 |  | Top of Form  unclassified Alphaproteobacteria  Bottom of Form | Top of Form  unclassified Alphaproteobacteria  Bottom of Form |
| SAR11 cluster alpha proteobacterium HIMB5 | 0 |  |  | Top of Form  unclassified Alphaproteobacteria  Bottom of Form | Top of Form  unclassified Alphaproteobacteria  Bottom of Form |

^a^ Strains with complete genomes (according to the MIST database, december 2014).

^b^ Number of genes encoding a sigma factor of the ECF15 family according to the MIST database.

^c^ Genes present at same locus as *phyR.*

^d^ These organisms possess two loci, each with one *ecfG* and one *phyR* gene.

^e^ The protein only consists of region σ_2_.

^f^ PhyR in this species only possesses the receiver domain.
