# Supplementary material for: Multiple σEcfG and NepR Proteins Are Involved in the General Stress Response in Methylobacterium extorquens
Source: PLoS One. 2016 Mar 30;11(3):e0152519. doi: 10.1371/journal.pone.0152519 (PMC4814048; doi:10.1371/journal.pone.0152519)
Supplement: S3 Table — (DOCX) [file pone.0152519.s007.docx]

S3 Table. σ^EcfG^ proteins of *Methylobacterium* species^a^.

| **Strain** | ***M. extorquens* AM1** | ***M. extorquens* DM4** | ***M. extorquens* CM4** | ***M. extorquens* PA1** | ***M. populi* BJ001** | ***M. radiotolerans* JCM2831** | ***Methylobacterium* sp. EUR3 AL-11** | ***Methylobacterium* sp. 4 46** | ***M. nodulans* ORS2060** | ***Methylobacterium* sp. 10** | ***Methylobacterium* sp. 77** | ***Methylobacterium* sp. 88A** |
| --- | --- | --- | --- | --- | --- | --- | --- | --- | --- | --- | --- | --- |
| **σ^EcfG^**  **proteins** | MexAM1_META1p4906 (σ^EcfG1^) | METDI_5498 | Mchl_4926 | Mext_4462 | Mpop_4976 | Mrad2831_1033 | K411DRAFT_6318 | M446_5269 | Mnod_5928 | K368DRAFT_1456 | A3OkDRAFT_3193 | A3OMDRAFT_3903 |
|  | MexAM1_META1p5327 (σ^EcfG2^) | METDI_5928 | Mchl_5355 | Mext_4870 | Mpop_5412 | Mrad2831_5909 | K411DRAFT_0365 | M446_5390 | Mnod_5581 | K368DRAFT_0542 | A3OKDRAFT_3918 | A3OMDRAFT_0123 |
|  | MexAM1_META1p0932 (σ^EcfG3^) | METDI1734 | MChl_1318 | Mext_1159 | Mpop_0268 | Mrad2831_1362 | K411DRAFT_5947 | M446_6692 | Mnod_7431 |  | A3OKDRAFT_0734 | A3OMDRAFT_0809 |
|  | MexAM1_META1p2698 (σ^EcfG4^) | METDI3311 | Mchl_2801 | Mext_2578 | Mpop_2606 | Mrad2831_2078 | K411DRAFT_5232 | M446_4500 | Mnod_5153 |  |  | A3OMDRAFT_2133 |
|  | MexAM1_META2p0154 (σ^EcfG5^) | METDI_1187 | Mchl_1057 | Mext_0458 | Mpop_4151 | Mrad2831_0903 | K411DRAFT_6451 | M446_1829 | Mnod_1217 |  |  |  |
|  | MexAM1_META2p1029 (σ^EcfG6^) |  |  | Mext_0132^c^ |  | Mrad2831_4026 | K411DRAFT_3147 | M446_0746 | Mnod_4833 |  |  |  |
|  |  |  |  |  |  | Mrad2831_5648 | K411DRAFT_1323 | M446_1277 |  |  |  |  |
|  |  |  |  |  |  |  |  | M446_0623 | Mnod_1554 |  |  |  |
|  |  |  |  |  |  |  |  | M446_6449 | Mnod_3118 |  |  |  |
|  |  |  |  |  |  |  |  | M446_6886 |  |  |  |  |
|  |  |  |  |  |  |  |  | M446_4689 |  |  |  |  |
| Total^b^ | 6 | 5 | 5 | 6 | 5 | 7 | 7 | 11 | 8 | 2 | 3 | 4 |

^a^ Only complete genomes or genomes represented by less than 10 contigs were considered.

^b^ Total number of ECF sigma factor of the σ^EcfG^ subfamily.

^c^ This σ^EcfG^ only possesses region σ_2._
