# Supplementary material for: Multiple σEcfG and NepR Proteins Are Involved in the General Stress Response in Methylobacterium extorquens
Source: PLoS One. 2016 Mar 30;11(3):e0152519. doi: 10.1371/journal.pone.0152519 (PMC4814048; doi:10.1371/journal.pone.0152519)
Supplement: S4 Table — (DOCX) [file pone.0152519.s008.docx]

| Strain or plasmid | Genotype or relevant feature | Reference |
| --- | --- | --- |
| **Strains** | | |
| ***E. coli***  DH5α | F^-^ *endA1 glnV44 thi-1 recA1 relA1 gyrA96 deoR nupG* φ80d*lacZ*ΔM15 Δ(*lacZYAargF*)  *U169 hsdR17^-^* | Invitrogen |
| TOP10 | F^-^ *mcrA* Δ(*mrr*-*hsdRMS*-*mcrBC*) φ80d*lacZ*ΔM15 Δ*lacX74 nupG recA1 araD139* _(*araleu*)*7697 galE15 galK16 rpsL* (Str^r^) *endA1* λ^−^ | Invitrogen |
| *dam*-/*dcm*- *E. coli* | ara-14 leuB6 fhuA31 lacY1 tsx78 glnV44 galK2 galT22 mcrA dcm-6 hisG4 rfbD1 R(zgb210::Tn10) Tet^S^ endA1 rspL136 (Str^R^) dam13::Tn9 (Cam^R^) xylA-5 mtl-1 thi-1 mcrB1 hsdR2 | NEB |
| BTH101 | F^-^ *cya-99 araD139 galE15 galK16 rpsL1 hsdR2 mcrA1 mcrB1* | Euromedex |
| ***M. extorquens* AM1** |  |  |
| JVZ958 | Wild-type strain | Lab strain |
| JVZ902 | Δ*ecfG1* (MexAM1_META1p4906) | {Francez-Charlot, 2009 #386} |
| JVZ1071 | Δ*ecfG4* (MexAM1_META1p2698) | This study |
| JVZ1073 | Δ*ecfG2* (MexAM1_META1p5327) | This study |
| JVZ1074 | Δ*ecfG3* (MexAM1_META1p0932) | This study |
| JVZ1157 | Δ*ecfG5* (MexAM1_META2p0154) | This study |
| JVZ1732 | Δ*ecfG6* (MexAM1_META2p1029) | This study |
| JVZ1163 | Δ*ecfG5* Δ*ecfG1* Δ*ecfG3* Δ*ecfG4* | This study |
| JVZ1182 | Δ*ecfG5* Δ*ecfG1* Δ*ecfG3* Δ*ecfG4* Δ*ecfG5* | This study |
| JVZ1183 | Δ*ecfG5* Δ*ecfG1* Δ*ecfG3* Δ*ecfG4* Δ*ecfG5* Δ*ecfG6* | This study |
| JVZ1391 | Δ*ecfG1* Δ*ecfG2* | This study |
| JVZ2182 | Δ*ecfG3* Δ*ecfG4* Δ*ecfG5* Δ*ecfG6* | This study |
| JVZ2183 | Δ*ecfG3* Δ*ecfG4* Δ*ecfG5* Δ*ecfG6* Δ*ecfG1* | This study |
| JVZ2185 | Δ*ecfG3* Δ*ecfG4* Δ*ecfG5* Δ*ecfG6* Δ*ecfG2* | This study |
| SM1 | Δ*ecfG3* Δ*ecfG4* Δ*ecfG5* Δ*ecfG6* Δ*ecfG1* | This study |
| SM2 | Δ*ecfG3* Δ*ecfG4* Δ*ecfG5* Δ*ecfG6* Δ*ecfG2* | This study |
| **Plasmids** | | |
| **Plasmids for mutagenesis** | | |
| pCM433 | Broad-host-range sacB-based allelic exchange vector; Ap^r^, Cm^r^, Tc^r^ | {Marx, 2008 #1064} |
| pK18mobsacB | Broad-host-range sacB-based allelic-exchange vector; Km^r^ | {Schafer, 1994 #1157} |
| pCM433-ecfG2 | pCM433 with fused up- and downstream regions of *ecfG2*; Ap^r^, Cm^r^, Tc^r^ | This study |
| pCM433- ecfG3 | pCM433 with fused up- and downstream regions of *ecfG3*; Ap^r^, Cm^r^, Tc^r^ | This study |
| pCM433-ecfG4 | pCM433 with fused up- and downstream regions of *ecfG4*; Ap^r^, Cm^r^, Tc^r^ | This study |
| pCM433-ecfG6 | pCM433 with fused up- and downstream regions of *ecfG6*; Ap^r^, Cm^r^, Tc^r^ | This study |
| pK18-0154 | pK18mobsacB with fused up- and downstream regions of ecfG5; Km^r^ | This study |
| **Plasmids for expression** | | |
| pCM62 | Broad-host-range cloning vector; IncP origin of replication; Tc^r^ | {Marx, 2001 #464} |
| pCM62HA | pCM62 derivative for generation of C-terminally HA tagged proteins; Tc^r^ | This study |
| pCM80 | Broad-host-range cloning vector; IncP origin of replication; *M. extorquens* AM1 expression vector (*mxaFp*); Tc^r^ | {Marx, 2001 #464} |
| pCM80_3xF | pCM80 derivative for generation of C-terminally triple flag-tagged proteins; Tc^r^ | This study |
| pCM62_ecfG1 | pCM62 with *ecfG1* ORF and upstream region; Tc^r^ | This study |
| pCM62_ecfG2 | pCM62 with *ecfG2* ORF and upstream region; Tc^r^ | This study |
| pCM62_ecfG3 | pCM62 with *ecfG3* ORF and upstream region; Tc^r^ | This study |
| pCM62_ecfG4 | pCM62 with *ecfG4* ORF and upstream region; Tc^r^ | This study |
| pCM62_ecfG5 | pCM62 with *ecfG5* ORF and upstream region; Tc^r^ | This study |
| pCM62_ecfG6 | pCM62 with *ecfG6* ORF and upstream region; Tc^r^ | This study |
| pCM62_ecfG1v1 | pCM62 with ecfG1 ORF and upstream region with first start codon mutated; Tc^r^ | This study |
| pCM62_ecfG1v2 | pCM62 with ecfG1 ORF and upstream region with second start codon mutated; Tc^r^ | This study |
| pCM62HA_ecfG1 | pCM62HA with *ecfG1* ORF and upstream region; Tc^r^ | This study |
| pCM62HA_ecfG2 | pCM62HA with *ecfG2* ORF and upstream region; Tc^r^ | This study |
| pCM62HA_ecfG3 | pCM62HA with *ecfG3* ORF and upstream region; Tc^r^ | This study |
| pCM62HA_ecfG4 | pCM62HA with *ecfG4* ORF and upstream region; Tc^r^ | This study |
| pCM62HA_ecfG5 | pCM62HA with *ecfG5* ORF and upstream region; Tc^r^ | This study |
| pCM62HA_ecfG6 | pCM62HA with *ecfG6* ORF and upstream region; Tc^r^ | This study |
| pCM80-ecfG1 | pCM80 with *ecfG1* ORF and upstream region; Tc^r^ | This study |
| pCM80-ecfG2 | pCM80 with *ecfG2* ORF and upstream region; Tc^r^ | This study |
| pCM80-ecfG3 | pCM80 with *ecfG3* ORF and upstream region; Tc^r^ | This study |
| pCM80-ecfG4 | pCM80 with *ecfG4* ORF and upstream region; Tc^r^ | This study |
| pCM80-ecfG5 | pCM80 with *ecfG5* ORF and upstream region; Tc^r^ | This study |
| pCM80-ecfG6 | pCM80 with *ecfG6* ORF and upstream region; Tc^r^ | This study |
| pCM80-735 | pCM80 with META2_0735 ORF and upstream region; Tc^r^ | This study |
| pCM80-1275 | pCM80 with META1_1275 ORF and upstream region; Tc^r^ | This study |
| pCM80-2700 | pCM80 with META1_2700 ORF and upstream region; Tc^r^ | This study |
| pCM80-nepR | pCM80 with *nepR* ORF and upstream region; Tc^r^ | {Francez-Charlot, 2009 #386} |
| pCM80_3xF-735 | pCM80_3xF with META2_0735 ORF and upstream region; Tc^r^ | This study |
| pCM80_3xF-1275 | pCM80_3xF with META1_1275 ORF and upstream region; Tc^r^ | This study |
| pCM80_3xF-2700 | pCM80_3xF with META1_2700 ORF and upstream region; Tc^r^ | This study |
| pCM80_3xF-nepR | pCM80_3xF with nepR ORF and upstream region; Tc^r^ | This study |
| **Plasmids for bacterial two-hybrid** | | |
| pKNT25 | Low-copy-number plasmid for BACTH, encoding the Bordetella pertussis CyaA T25 fragment for fusions to its N terminus; Km^r^ | Euromedex |
| pUT18 | High-copy-number plasmid for BACTH, encoding the Bordetella pertussis CyaA T18 fragment for fusions to its N terminus; Ap^r^ | Euromedex |
| pKNT25-4906FL | pKNT25 with *ecfG1* ORF; Km^r^ | This study |
| pKNT25-4906S | pKNT25 with N-terminally truncated *ecfG1* ORF; Km^r^ | This study |
| pKNT25-5327FL | pKNT25 with *ecfG2* ORF; Km^r^ | This study |
| pKNT25-5327S | pKNT25 with N-terminally truncated *ecfG2* ORF; Km^r^ | This study |
| pKNT25-932FL | pKNT25 with *ecfG3* ORF; Km^r^ | This study |
| pKNT25-932S | pKNT25 with N-terminally truncated *ecfG3* ORF; Km^r^ | This study |
| pKNT25-2698FL | pKNT25 with *ecfG4* ORF; Km^r^ | This study |
| pKNT25-2698S | pKNT25 with N-terminally truncated *ecfG4* ORF; Km^r^ | This study |
| pKNT25-154FL | pKNT25 with *ecfG5* ORF; Km^r^ | This study |
| pKNT25-154S | pKNT25 with N-terminally truncated *ecfG5* ORF; Km^r^ | This study |
| pKNT25-1029FL | pKNT25 with *ecfG6* ORF; Km^r^ | This study |
| pKNT25-1029S | pKNT25 with N-terminally truncated *ecfG6* ORF; Km^r^ | This study |
| pUT18-735 | pUT18 with MexAM1_META2p0735 ORF; Ap^r^ | This study |
| pUT18-1275 | pUT18 with MexAM1_META1p1275 ORF; Ap^r^ | This study |
| pUT18-2700 | pUT18 with MexAM1_META1p2700 ORF; Ap^r^ | This study |
| pUT18-nepR | pUT18 with *nepR* ORF; Ap^r^ | This study |
| **Plasmids for transcriptional fusions** | | |
| pLM05 | Plasmid for generation of transcriptional fusion with *luxCDABE* operon; Km^r^ | {Metzger, 2013 #1226} |
| pLM05-735p | pLM05 with MexAM1_META2p0735 promoter region; Km^r^ | This study |
| pLM05-1275p | pLM05 with MexAM1_META1p1275 promoter region; Km^r^ | This study |
| pLM05-2700p | pLM05 with MexAM1_META1p2700 promoter region; Km^r^ | This study |
| pLM05-ecfG1p | pLM05 with *ecfG1* promoter region; Km^r^ | This study |
| pLM05-ecfG2p | pLM05 with *ecfG2* promoter region; Km^r^ | This study |
| pLM05-ecfG3p | pLM05 with *ecfG3* promoter region; Km^r^ | This study |
| pLM05-ecfG4p | pLM05 with *ecfG4* promoter region; Km^r^ | This study |
| pLM05-ecfG5p | pLM05 with *ecfG5* promoter region; Km^r^ | This study |
| pLM05-ecfG6p | pLM05 with *ecfG6* promoter region; Km^r^ | This study |
| pLM05-nepRp | pLM05 with *nepR* promoter region; Km^r^ | This study |
| pLM12 | pLM05 with MexAM1_META1p1696 promoter region; Km^r^ | {Metzger, 2013 #1226} |
