# Supplementary material for: Multiple σEcfG and NepR Proteins Are Involved in the General Stress Response in Methylobacterium extorquens
Source: PLoS One. 2016 Mar 30;11(3):e0152519. doi: 10.1371/journal.pone.0152519 (PMC4814048; doi:10.1371/journal.pone.0152519)
Supplement: S5 Table — (DOCX) [file pone.0152519.s009.docx]

| Plasmid | Primer names | Sequences |
| --- | --- | --- |
| pCM433-ecfG2 | 5327_up_F  5327_up_R  5327_dw_F  5327_dw_R | ATAACATATGCCCATCACGTCGGAGAGAAAC  TTAGGCCATCCACGTCTTTGGCATCAGGTCCTGTGGCTG  ATGCCAAAGACGTGGATGGCCTAAAGCGCGGCCCG  AATACCGGTTTGATGGCCTCGGAGG |
| pCM433- ecfG3 | 932_up_F2  932_up_R2  932_dw_F2  932_dw_R | ATAACATATGCCACCTCAATCCGCTTC  TCAGGCCGGCACAGCATGTCGATCAACCCACGTTCCTTC  GATCGACATGCTGTGCCGGCCTGAACTTCATCTTTCTCC  AATACCGGTACCGGCTCTGGGCCGATGCCCTG |
| pCM433-ecfG4 | 2698_up_F  2698_up_R2  2698_dw_F2  2698_dw_R | AAACATATGCCGTCCTGCACCTTGGATGACAGC  TCAGGCCCGTGCTTCGTCGGGCATCCGAAAGGGC  ATGCCCGACGAAGCACGGGCCTGACCCGCGC  TTATGCGGCCGCTTCTCCGATCATTCTTGC |
| pCM433-ecfG6 | 1029_up_F  1029_up_R2  1029_dw_F2  1029_dw_R | ATAACATATGGCGTTCGAGGTCGACGACGATCG  TCAAGCAAGTGCGCTCTCGCCCATCGGTCGTGATCCGC  ATGGGCGAGAGCGCACTTGCTTGATGCGAGATCCGCC  TTATGCGGCCGCCTGAGGTGCAGCG |
| pCM62_ecfG1 | 4906-his_prom_f  4906_r4_r | TATATA TCTAGA TGAGGGCGATCGATCGGGAAAGG  tatataggatccTCAGGCGTCGGGCATCGCCGACTG |
| pCM62_ecfG1v1 | 4906-his_prom_f  4906 start1 F  4906 start1 R  4906_r4_r | TATATA TCTAGA TGAGGGCGATCGATCGGGAAAGG  GAGCCCGTAGCGTAACGATACC  GGTATCGTTACGCTACGGGCTC  tatataggatccTCAGGCGTCGGGCATCGCCGACTG |
| pCM62_ecfG1v2 | 4906-his_prom_f  4906 start2 F  4906 start2 R  4906_r4_r | TATATA TCTAGA TGAGGGCGATCGATCGGGAAAGG  CCCGCGATCACAGACGGCC  GGCCGTCTGTGATCGCGGG  tatataggatccTCAGGCGTCGGGCATCGCCGACTG |
| pCM62_ecfG2 | 5327comp_F  5327comp_R | ATT TTG CAT GCC TCG TTC GAC GCG ACT GG  ATT TTG GTA CCT TAG GCC ATC CAG GTC G |
| pCM62_ecfG3 | 0932comp_F  0932comp_R | ATT TTA AGC TTC GAC GAC CGC GAA CCG ACC  ATT TTT CTA GAT CAG GCC GGC ACC TTG |
| pCM62_ecfG4 | 2698comp_F  2698comp_R | ATT TTG CAT GCT CTT CAG CCG GCC TCA TAC C  ATT TTG GTA CCA TGC GGC TGC CCA GAG |
| pCM62_ecfG5 | 0154comp_F  0154comp_R | ATT TTG CAT GCT CGG GCG GAA TGC TTG ATG C  ATT TTT CTA GAT CAA AGC GCA GTG CTG C |
| pCM62_ecfG6 | 1029comp_F  1029comp_R | ATT TTG CAT GCC GGG CGT GGT CGG GCT TC  ATT TTG GTA CCT CAA GCA AGT GCA TCG |
| pCM62HA | HA_s  HA_as | gtacCTACCCGTACGACGTCCCGGACTACGCCTGATGAG  aattCTCATCAGGCGTAGTCCGGGACGTCGTACGGGTAG |
| pCM62HA_ecfG1 | 4906comp_F  4906 no stop R | ATT TTG CAT GCC GCA TTC AAG CGA CAG G  ATTTTGGTACCGGCGTCGGGCATCGCCGACTGG |
| pCM62HA_ecfG2 | 5327comp_F  5327 wo stop | ATT TTG CAT GCC TCG TTC GAC GCG ACT GG  atttggtaccGGCCATCCAGGTCGAGCC |
| pCM62HA_ecfG3 | 0932comp_F  932 wo stop | ATT TTA AGC TTC GAC GAC CGC GAA CCG ACC  atttggtaccGGCCGGCACCTTGGTGG |
| pCM62HA_ecfG4 | 2698comp_F  2698 wo stop | ATT TTG CAT GCT CTT CAG CCG GCC TCA TAC C  atttggtaccGGCCCGTGCCGCCGACAATC |
| pCM62HA_ecfG5 | 0154comp_F  154 wo stop | ATT TTG CAT GCT CGG GCG GAA TGC TTG ATG C  Atttggatcc AAGCGCAGTGCTGCGCGTG |
| pCM62HA_ecfG6 | 1029comp_F  1029 wo stop | ATT TTG CAT GCC GGG CGT GGT CGG GCT TC  atttggtaccAGCAAGTGCATCGGTAGACG |
| pCM80-735 | 735 F1  735 R1 | GAC TAA GCT TAC CTT GGC CGT TTC ATT TGT GC  GAC TTC TAG ATG TGA CGG GCT GCC CTC AG |
| pCM80-1275 | 1_1275 F HindIII  1_1275 R XbaI | GACTAAGCTTCCTCACAGCCGCTTCC  GACTTCTAGAAGCCTCAGTCGCGGGTC |
| pCM80-2700 | 1_2700 F HindIII  1_2700 R XbaI | GACTAAGCTTGATGTTTGCGCCTGGAC  GACTTCTAGACCTACTTCTTCGGATCGC |
| pCM80-ecfG1 | 4906_F_HindIII  4906_R_XbaI | AAGCTTCGATCGCGAAGAGCCCGATGCG  TCTAGACGGTTCCGGCGTCCTTCGAGC |
| pCM80-ecfG2 | 1_5327 F PstI  1_5327 R XbaI | CTG CAG GTG ACC AGC CAC AGG AC  TCT AGA TTA GGC CAT CCA GGT CG |
| pCM80-ecfG3 | 1_0932 F XbaI  1_0932 R XbaI | TCT AGA CTC TGC CGC TCG CTC C  TCT AGA TCA GGC CGG CAC CTT G |
| pCM80-ecfG4 | 1_2698 F PstI  1_2698 R XbaI | CTG CAG ACG CCC TGC CCT ATC C  TCT AGA ATG CGG CTG CCC AGA G |
| pCM80-ecfG5 | 2_0154 F1 XbaI  2_0154 R1 XbaI | TCT AGA GGG AGG CGA AAG GGA CAG  TCT AGA TCA AAG CGC AGT GCT GC |
| pCM80-ecfG6 | 2_1029 F2 PstI  2_1029 R2 XbaI | AAA ACT GCA GCG ATG AAC CAC TTC AGT G  CTA GTC TAG ATC AAG CAA GTG CAT CG |
| pCM80_3xF | Flag F  Flag R | ATTTTGGTACCGACTACAAGGACCACGACG  ATTTTGAATTCTCATCACTTGTCGTCGTCGTCC |
| pCM80_3xF-735 | 735 F1  735 wo stop | GACTAAGCTTACCTTGGCCGTTTCATTTGTGC  atttggtaccGGCGGACGCGGAACCGCC |
| pCM80_3xF-1275 | 1_1275 F HindIII  1275 wo stop | GACTAAGCTTCCTCACAGCCGCTTCC  atttggtaccGTCGCGGGTCGCCCGGTC |
| pCM80_3xF-2700 | 1_2700 F HindIII  2700 wo stop | GACTAAGCTTGATGTTTGCGCCTGGAC  atttggtaccCTTCTTCGGATCGCCCGC |
| pCM80_3xF-nepR | 3109 80 F  3109 wo stop | attttaagcttAGCCATGACTCATGTCAAGC  atttggtaccGACGCTCGCCGGATCGTC |
| pK18-0154 | 0154_uppk_F  0154_up_R2  0154_dw_F2  0154_downpk_R | TATCTGCAGCCTCGAACACGACGTGC  TCAAAGCGCAGTTCCTTCGGCCATGGCTGTCCCTTTC  ATGGCCGAAGGAACTGCGCTTTGAGCTGCCCGTTCC  ATAGTCGACATGGCCGTGCAGGAGCCGG |
| pKNT25-4906FL | 4906 F bacth XbaI  4906 R bacth acc65I | atttttctagagATGCGTAACGATACCGAAG  attttggtacccgGGCGTCGGGCATCGCCGACTGG |
| pKNT25-4906S | 4906 F S bacth  4906 R bacth acc65I | atttttctagagGGCGAGCGGATCGATC  attttggtacccgGGCGTCGGGCATCGCCGACTGG |
| pKNT25-5327FL | 5327 F bac  5327 R bac | atttttctagagATGCCAAAGACGACCCTCG  attttggtacccgGGCCATCCAGGTCGAGCC |
| pKNT25-5327S | 5327woN F bac  5327 R bac | atttttctagagGAAGGGAAGGACGAGGAATCG  attttggtacccgGGCCATCCAGGTCGAGCC |
| pKNT25-932FL | 932 F bac  932 R bac | atttttctagag TTGATCGACATGCTGATTGC  attttggtacccg GGCCGGCACCTTGGTGGAAG |
| pKNT25-932S | 932 woN F bac  932 R bac | atttttctagag GCGAGTGAGCGCGACGCAGC  attttggtacccg GGCCGGCACCTTGGTGGAAG |
| pKNT25-2698FL | 2698 F bac  2698R bac | atttttctagag ATGCCCGACGAACTGATTCG  attttggtacccg GGCCCGTGCCGCCGACAATC |
| pKNT25-2698S | 2698 woN F bac  2698R bac | atttttctagagCATGGCGGAGCGGTCGAAACC  attttggtacccg GGCCCGTGCCGCCGACAATC |
| pKNT25-154FL | 154 F bac  154 R bac | atttttctagagATGGCCGAAGGAACTTCC  attttggtacccg AAGCGCAGTGCTGCGCGTG |
| pKNT25-154S | 154 woN F bac  154 R bac | AtttttctagagAGGGAGGCTGCAGACTTCC  attttggtacccg AAGCGCAGTGCTGCGCGTG |
| pKNT25-1029FL | 1029 F bac  1029 R bac | atttttctagag ATGGGCGAGAGCTTTCCC  attttggtacccg AGCAAGTGCATCGGTAGAC |
| pKNT25-1029S | 1029 woN F bac  1029 R bac | atttttctagag AAAAGCCGTGACGACGACG  attttggtacccg AGCAAGTGCATCGGTAGAC |
| pLM05-735p | 0735p_F2  0735p_R2 | attttactagtcatccggcgctgcccgaac  attttccatggacgcgagcccttgccttcag |
| pLM05-1275p | 1275p_F2  1275p_R2 | attttactagtctctggctgccggacctgc  attttccatggggcttcgtcacggatcgtctc |
| pLM05-2700p | 2700p_F2  2700p_R2 | attttactagtccactgccggggcctttttc  attttccatgggttttcggcggatgtgtcggag |
| pLM05-ecfG1p | 4906p1_F2  4906p_R3 | attttactagtgccgcattcaagcgacagg  attttccatggatcgttacgcatcgggctcttc |
| pLM05-ecfG2p | 5327p_F2  5327p_R2 | attttactagtctcgttcgacgcgactgg  attttccatgggtctttggcatcaggtcctgtg |
| pLM05-ecfG3p | 0932p_F2  0932p_R2 | attttactagtcgacgaccgcgaaccgacc  attttccatggcggaccgagtccgcttgcaatc |
| pLM05-ecfG4p | 2698p_F2  2698p_R2 | attttactagttcttcagccggcctcatacc  attttccatggatccggcaattcactgcgaatc |
| pLM05-ecfG5p | 0154p_F2  0154p_R2 | attttactagttcgggcggaatgcttgatgc  attttccatggagctgtccgggaagttccttcg |
| pLM05-ecfG6p | 1029p_F2  1029p_R2 | attttactagtcgggcgtggtcgggcttc  attttccatggagctctcgcccatcggtcgtg |
| pLM05-nepRp | 3109p_F2  3109p_R2 | attttactagtataacggcgcagatacgg  attttccatggctcattcatgcgttcaagatcc |
| pUT18-735 | 735 F bac  735 R bac | atttttctagag ATGACCGCTGAAGGCAAGG  attttggtacccg GGCGGACGCGGAACCGCCC |
| pUT18-1275 | 1275 F bac  1275 R bac | atttttctagag GTGACGAAGCCCATCGCC  attttggtacccg GTCGCGGGTCGCCCGGTCC |
| pUT18-2700 | 2700 F bac  2700 R bac | atttttctagag ATGACCGGCTCCGACACATCC  attttggtacccg CTTCTTCGGATCGCCCGC |
| pUT18-nepR | nepR F2 bacth  nepR R bacth | atttttctagagATGAATGAGAACGAGCCG  attttggtacccgGACGCTCGCCGGATCGTC |
